# Efficient Suppression of Hepatitis C Virus Replication by Combination Treatment with miR-122 Antagonism and Direct-acting Antivirals in Cell Culture Systems

**DOI:** 10.1038/srep30939

**Published:** 2016-08-03

**Authors:** Fanwei Liu, Tetsuro Shimakami, Kazuhisa Murai, Takayoshi Shirasaki, Masaya Funaki, Masao Honda, Seishi Murakami, Minkyung Yi, Hong Tang, Shuichi Kaneko

**Affiliations:** 1 Department of Gastroenterology, Kanazawa University Hospital, Kanazawa, Ishikawa 920-8641, Japan; 2 Department of Microbiology and Immunology, University of Texas Medical Branch at Galveston, Galveston, TX 77555-0144, USA; 3 Center of Infectious Diseases, West China Hospital, Sichuan University, Chengdu 610041, China

## Abstract

Direct-acting antivirals (DAAs) against Hepatitis C virus (HCV) show effective antiviral activity with few side effects. However, the selection of DAA-resistance mutants is a growing problem that needs to be resolved. In contrast, miR-122 antagonism shows extensive antiviral effects among all HCV genotypes and a high barrier to drug resistance. In the present study, we evaluated three DAAs (simeprevir, daclatasvir, and sofosbuvir) in combination with anti-miR-122 treatment against HCV genotype 1a in cell cultures. We found that combination treatments with anti-miR-122 and a DAA had additive or synergistic antiviral effects. The EC_50_ values of simeprevir in simeprevir-resistant mutants were significantly decreased by combining simeprevir with anti-miR-122. A similar reduction in EC_50_ in daclatasvir-resistant mutants was achieved by combining daclatasvir with anti-miR-122. Combination treatment in HCV-replicating cells with DAA and anti-miR-122 sharply reduced HCV RNA amounts. Conversely, DAA single treatment with simeprevir or daclatasvir reduced HCV RNA levels initially, but the levels later rebounded. DAA-resistant mutants were less frequently observed in combination treatments than in DAA single treatments. In summary, the addition of miR-122 antagonism to DAA single treatments had additive or synergistic antiviral effects and helped to efficiently suppress HCV replication and the emergence of DAA-resistant mutants.

About 170 million people worldwide are currently infected with hepatitis C virus (HCV)^1^ and approximately 500,000 people die each year from HCV-related liver diseases, such as liver failure and hepatocellular carcinoma^2^. The previous standard of care for HCV was based on PEGylated interferon and ribavirin therapy, which eliminated HCV in about 50% of patients infected with genotype 1 HCV.

The establishment of HCV cell culture systems has facilitated the development of direct-acting antiviral reagents (DAAs) targeting the NS3/4A protease, NS5A protein, and NS5B polymerase. The combination of a DAA such as NS3/4A protease inhibitor, NS5A inhibitor, or nucleotide analog NS5B polymerase inhibitor, with PEGylated interferon and ribavirin has improved sustained virologic response rates compared with the standard of care. However, even a DAA combined with PEGylated interferon and ribavirin, it remains difficult to treat patients with a poor response to interferon (i.e., null responders and non-CC IL28B)^3^,^4^,^5^. In addition, interferon-based therapies have a number of severe adverse effects. Recently, many combination regimens involving DAAs without the use of PEGylated interferon, called interferon-free DAA therapies, have been reported to dramatically increase the rate of sustained virologic responses. Interferon-free DAA therapies are generally accompanied by fewer adverse effects and have a shorter treatment period than interferon-based therapies^6^,^7^. However, the pre-existence and selection of resistance-associated variants (RAVs) can result in treatment failure^8^. Furthermore, RAVs may remain in the liver for long periods after treatment once selected by DAA therapies; NS5A inhibitor RAVs are particular examples^9^. RAVs can also interfere with subsequent interferon-free DAA therapies because there are a limited number of DAA classes and RAVs sometimes show cross-drug resistance to the same class. Therefore, a new strategy to both prevent the emergence of DAA-resistant mutants and effectively eliminate pre-existing RAVs is urgently needed.

In addition, apart from the nucleotide analog NS5B polymerase inhibitor sofosbuvir^10^,^11^, most DAAs show different antiviral activities against different HCV genotypes. Therefore, new classes of antiviral therapies applicable to all HCV genotypes, known as pan-genotypic antiviral therapies, are also needed.

One liver-specific microRNA (miRNA), miR-122, which is abundant in the liver, binds two sites within the 5′untranslated region (UTR) of the viral genome, and recruits the argonaute 2 protein^12^,^13^,^14^. Through this direct interaction with the HCV genome, miR-122 increases HCV genome stability, translation, and amplification, which results in increased HCV replication, as discussed in a recent review article^15^. Hence, sequestration of miR-122 by anti-miR-122 oligonucleotides could be a valid treatment strategy, as has been proven not only in HCV cell culture systems, but also in HCV-infected chimpanzees and humans^16^,^17^. Anti-miR-122 therapy using anti-miR-122 oligonucleotides has a number of advantages over interferon-free DAA therapies. First, anti-miR-122 therapy is expected to show universal antiviral effects on all HCV genotypes because miR-122 binding sites at the 5′ UTR are highly conserved among all HCV genotypes^18^. Second, it provides a high barrier to the emergence of resistance because miR-122–binding negative HCV mutants are highly unfit in HCV cell culture systems^19^. Miravirsen (SPC3649) is a 15-base oligonucleotide with locked nucleic acid (LNA) modification complementary to part of miR-122. It is one of the most developed anti-miR-122 therapies and has been intravenously administered to African green monkeys^20^. Miravirsen is taken up by hepatocytes and forms stable heteroduplexes with miR-122, resulting in efficient depletion of free miR-122. When miravirsen was administered to HCV-infected chimpanzees, it effectively suppressed HCV RNA levels without any evidence of resistant mutants^17^. In a phase 2a clinical study, miravirsen administered to HCV-infected patients exerted substantial and prolonged dose-dependent reductions in HCV RNA levels without any escape mutants^16^. However, it might be difficult to completely eliminate HCV from infected cells via anti-miR-122 therapy alone because HCV can replicate within Hela cells, which do not express miR-122^21^.

In summary, DAAs can effectively suppress HCV replication and have a low barrier to the emergence of DAA-resistant viruses. Conversely, anti-miR-122 therapy has a moderate antiviral activity with a high barrier to drug resistance. In this study, we explored whether these two antiviral therapies could compensate for each other’s weaknesses and effectively suppress HCV replication in HCV cell culture systems.

## Results

### Antiviral Effects of DAAs and LNA–anti-miR-122

**Figure 1:**
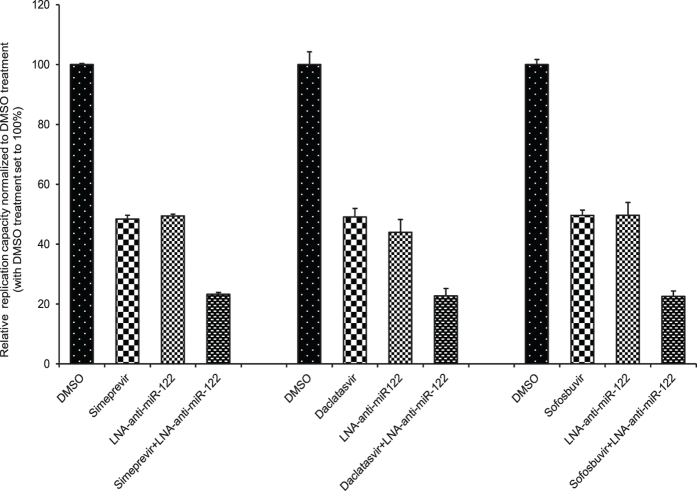
The potential additive or synergistic antiviral effects of LNA–anti-miR-122 and DAA treatment. H77S.3/GLuc2A RNA was transfected into FT3-7 cells and then treated with DMSO, LNA–anti-miR-122, or LNA–anti-miR-122 plus DAA. The concentrations of the antiviral drugs (simeprevir, daclatasvir, sofosbuvir, and LNA–anti-miR-122) were set to their EC_50_ values. GLuc activity secreted by H77S.3/GLuc2A RNA-transfected cells was determined and normalized to that of the control group. Results are shown as the means + SEM.

Although LNA–anti-miR-122 has been added to the cell culture medium in previous studies^22^, we transfected it into cells because transfection is a more effective way to suppress HCV replication^14^. To evaluate the transfection efficiency of single-stranded RNA oligonucleotides, we transfected 10 nM FAM-labeled 2′-*O*-methyl (2′OMe) single-stranded RNA, anti-miR-122 2′OMe^12^ into Huh-7.5 cells using siPORT NeoFX Transfection Agent and fluorescence was detected by fluorescence microscopy. As shown in Supplementary Fig. S1, almost all cells showed fluorescence from FAM-labeled anti-miR-122 2′OMe transfected cells. To exclude the potential of LNA-anti-miR-122 cytotoxicity, we evaluated the CC_50_ values of LNA-anti-miR-122 for naïve FT3-7 cells, Huh-7.5 cells, and Huh-7.5 cells harboring a full-genome HCV replicon. The CC_50_ of LNA-anti-miR-122 was more than 100 nM in the cells tested (Supplementary Fig. S2a–c). First, we determined the EC_50_ values of LNA–anti-miR-122, simeprevir, an inhibitor of NS3/4A, daclatasvir, an inhibitor of NS5A, and sofosbuvir, an inhibitor of the nucleotide analog for genotype 1a H77S.3/GLuc2A^23^ in Huh-7.5 cells to be about 10 nM, 2 nM, 10 pM, and 50 nM, respectively. Transfection of single-stranded RNA may have potential antiviral effect. To exclude this possibility, we tested the effects of a non-relevant single-stranded RNA oligonucleotide as the LNA-RNA-Control on HCV replication. We did not observe any significant effects in LNA-RNA-Control on HCV replication (Supplementary Fig. S3). We next examined whether the combination of a DAA and LNA–anti-miR-122 could suppress HCV replication more efficiently than a single DAA. In this experiment, H77S.3/GLuc2A-replicating cells were treated with a DAA (simeprevir, daclatasvir, or sofosbuvir) and/or LNA–anti-miR-122 at their EC_50_ values. When HCV-replicating cells were treated with both DAA and LNA–anti-miR-122, the combination suppressed HCV replication approximately two-fold more efficiently than did either agent alone (Fig. 1). The degree of the additional antiviral effects of LNA–anti-miR-122 was comparable among the DAAs tested. These results strongly suggested that there could be additive or synergistic antiviral effects in combination therapy with a DAA and LNA–anti-miR-122.

### *In Vitro* Antiviral Activity in Drug-resistant HCV Mutants

Our results indicated that combination treatment with LNA–anti-miR-122 and a DAA had a potential synergistic or additive antiviral effect on wild-type H77S (Fig. 1). To confirm these potential effects, we examined combinations of LNA–anti-miR-122 with simeprevir, daclatasvir, or sofosbuvir. Briefly, we treated H77S.3/GLuc2A-replicating cells with various concentrations of LNA–anti-miR-122 and DAAs, and the antiviral activity was evaluated via GLuc activity. We then calculated the combination index by using the method of Chou and Talalay with CalcuSyn Software (Supplementary Table S1a–c)^24^. If the combination index was less than 0.8, the two treatments were assumed to have a synergistic effect, while if it was between 0.8 and 1.2 their effects were considered additive. A combination of simeprevir and LNA–anti-miR-122 showed a mostly synergistic antiviral effect (Supplementary Fig. S4a) and we obtained similar results with daclatasvir (Supplementary Fig. S4b) and sofosbuvir (Supplementary Fig. S4c). When we calculated the EC_50_ of simeprevir, daclatasvir, and sofosbuvir for wild-type virus in the presence of LNA–anti-miR-122, it was generally lower for all the DAAs tested, depending on the concentration of LNA–anti-miR-122 (Supplementary Fig. S4d–f). These data suggest that LNA–anti-miR-122 and DAAs combination treatments show synergistic or additive antiviral effect on HCV genotype 1a H77S. At that point, we did not know whether a similar antiviral effect would be observed in DAA-resistant mutants. Thus, we evaluated the antiviral activity of combination treatment against well-known DAA-resistant mutants, as summarized in a recent review^8^. We then tested the activity of the combination treatments for the mutants listed in Table 1 and Supplementary Table S2 (NS3 mutants) and those in Table 2 (NS5A mutants) in the H77S.3/GLuc2A backbone. For this purpose, we determined that the EC_50_ values of the DAAs for each mutant at each concentration of LNA–anti-miR-122. LNA–anti-miR-122 generally ameliorated drug resistance to simeprevir-resistant mutants (Table 1 and Supplementary Table S2). For example, in the presence of 0.5 nM LNA–anti-miR-122, the antiviral effects of simeprevir against resistance variants were greatly enhanced, and simeprevir susceptibility ranged from no change (V36G, F43S) to a 4.6-fold change (R155T). In comparison with cells not treated with LNA–anti-miR-122, changes were approximately 2-fold for most of the other mutants. Interestingly, we did not consistently observe a similar change in susceptibility among all combinations of the concentrations tested. For example, the EC_50_ values of simeprevir for the S122R and D168E mutants in the presence of 10 nM LNA–anti-miR-122 were comparable to those in the absence of LNA–anti-miR-122. We performed the same study in daclatasvir-resistant variants and observed a similar phenomenon (Table 2). In the presence of 1 nM LNA–anti-miR-122, the susceptibility of Q30R and L31V mutants to daclatasvir increased about 4.5-fold and 2.5-fold, respectively. The change in susceptibility was observed for any combination of concentrations, which differed from the results for the simeprevir-resistant mutants. We did not analyze another well-known daclatasvir-resistant mutant, Y93H in NS5A, because the fitness of this mutant was too severely impaired to evaluate the change in susceptibility^25^. We also omitted a well-known sofosbuvir-resistant mutant, S282T in NS5B, from our study due to its low capacity for replication^11^. In summary, the potency of DAAs against their corresponding DAA-resistant variants was significantly enhanced in the presence of LNA–anti-miR-122.

### *In Vitro* Selection of DAA-resistant Mutants with DAAs, LNA–anti-miR-122, or DAA plus LNA–anti-miR-122 in Genotype 1a H77S-replicating Cells

We anticipated that the synergistic or additive antiviral effects of LNA–anti-miR-122 combined with a DAA on HCV replication could prevent the emergence of DAA-resistant mutants, which are expected during and/or after treatment with a single DAA. In an *in-vitro* experiment for the efficient selection of drug-resistant mutants, we used a full-genomic bicistronic H77S replicon, tat/2A-Neo-H77S, which contains tat, FMDV2A, and a neomycin-resistant gene as a first cistron driven by HCV IRES, and HCV polyproteins as a second cistron driven by EMCV IRES. In Huh-7.5 cells maintained for a few weeks after transfection of H77S.3/GLuc2A RNA, we found that the numbers of the cells harboring HCV decreased over time (data not shown). Moreover, an advantage of using tat/2A-Neo-H77S under G418 selection is that cells harboring HCV replication can be continuously selected. HCV polyproteins from the core to NS5B driven by EMCV IRES, tat/2A-Neo-H77S RNA, were transfected into Huh-7.5 cells, and transfected cells were selected using 1 mg/mL G418 for 2 weeks. We confirmed that all the G418-resistant cells supported HCV replication via immunofluorescence analysis using anti-core antibody (Supplementary Fig. S5a). Cells were maintained in the presence of 1 mg/mL G418 and split twice a week, and 10 nM LNA–anti-miR-122 was transfected at each split in the LNA–anti-miR-122 and LNA–anti-miR-122 plus DAA groups. Fresh DAA was also added at each split in the DAA and LNA–anti-miR-122 plus DAA groups. During each split, total RNA was extracted for the quantification of HCV RNA and sequence analysis. Transfected LNA–anti-miR-122 continuously showed antiviral activity without a reduction in potency until at least 5 days after transfection. Neither the transfection reagent nor the LNA-RNA-Control showed any effects on HCV replication (Supplementary Fig. S6).

**Figure 2:**
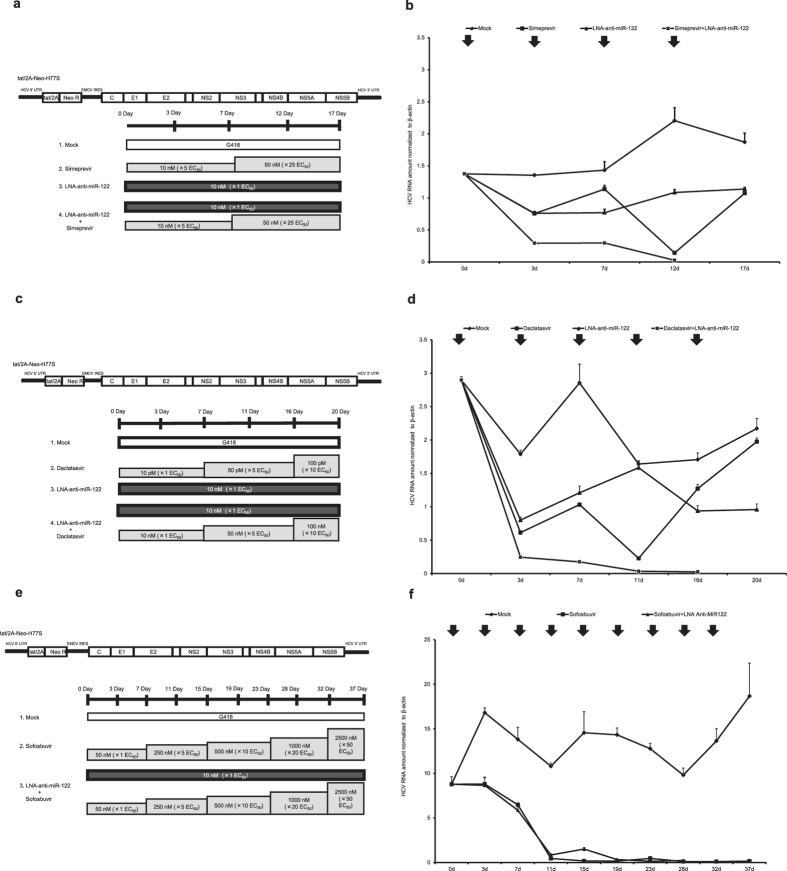
*In vitro* selection experiments in genotype 1a HCV-replicating cells. Huh-7.5 cells harboring tat/2A-Neo-H77S were treated with mock, DAA (simeprevir, daclatasvir or sofosbuvir), LNA–anti-miR-122, or DAA plus LNA–anti-miR-122 in the presence of G 418. The concentration of DAA was gradually increased, and LNA–anti-miR-122 was transfected into Huh-7.5 cells twice a week at cell passage. Schematic representations of the experiment using tat/2A-Neo-H77S-replicating cells with simeprevir (**a**), tat/2A-Neo-H77S-replicating cells with daclatasvir (**c**), and tat/2A-Neo-H77S-replicating cells with sofosbuvir (**e**). The amount of HCV RNA was normalized to that of β-actin mRNA in each group (**b**,**d**,**f**). The arrows in the upper of each figure represent the time points of LNA-anti-miR-122 transfection and DAA addition. Results are shown as means + SEM.

Cells harboring tat/2A-Neo-H77S were treated with either simeprevir or LNA–anti-miR-122. The concentration of simeprevir was started with 10 nM (5 × EC_50_). In simeprevir alone cells, the amount of HCV RNA gradually decreased until day 12, accompanied by cell death via G418 selection (Fig. 2a,b and Supplementary Fig. S7a). However, the amount of HCV RNA subsequently started to increase, which strongly suggested that simeprevir-resistant mutants were emerging. In the case of cells treated with 10 nM LNA–anti-miR-122 alone, the amount of HCV RNA decreased by day 7 and then reached a plateau, suggesting that LNA–anti-miR-122–resistant mutants were unlikely to emerge. In contrast, the amount of HCV RNA continuously decreased in cells treated with simeprevir plus LNA–anti-miR-122, and all cells were dead by day 12. Moreover, we obtained the same result when we performed a similar experiment in Huh-7.5 cells harboring H77S.3/Blast5A, which has a Blasticidin S-coding resistance gene within NS5A (Supplementary Result 2, Supplementary Figs S5b and S7b and S8a,b).

We performed a similar study in Huh-7.5 cells harboring tat/2A-Neo-H77S by treating them with either daclatasvir or LNA–anti-miR-122. First, we also started daclatasvir with the concentration of 50 pM (5 × EC_50_) as we did simeprevir, however, all cells died even in a daclatasvir single treatment group (data not shown). Thus, we decreased the starting concentration of daclatasvir to 10 pM (1 × EC_50_). In the cells treated with only daclatasvir, the amount of HCV RNA gradually decreased until day 11 (Fig. 2c,d and Supplementary Fig. S7c). However, the level began to increase thereafter, strongly suggesting that daclatasvir-resistant mutants were emerging. In cells treated with 10 nM LNA–anti-miR-122 alone, the amount of HCV RNA decreased by day 3 and then reached a plateau, suggesting that LNA–anti-miR-122–resistant mutants were unlikely to emerge. In contrast, the amount of HCV RNA continuously decreased in the cells treated with daclatasvir plus LNA–anti-miR-122, and resulted in the death of all cells by day 16.

We performed the same *in vitro* experiment using sofosbuvir as the simeprevir and daclatasvir experiments. First, we started sofosbuvir with the concentration of 250 nM (5 × EC_50_), however, all cells died even in a sofosbuvir treatment group (data not shown). Thus, we decreased the starting concentration of sofosbuvir to 50 nM (1 × EC_50_). We treated the cells with sofosbuvir or sofosbuvir plus LNA–anti-miR-122 and gradually increased the sofosbuvir concentration. HCV RNA levels sharply decreased in both the sofosbuvir single and combination treatment groups until day 11 (Fig. 2e,f and Supplementary Fig. S7d). All cells in the combination treatment group died by day 28. We did not observe HCV rebound during our study in the sofosbuvir single treatment group, in contrast to the simeprevir and daclatasvir experiments. Interestingly, the cells treated with sofosbuvir alone still grew with a low level of HCV RNA, while all cells died in the combination treatment group (Fig. 2f). We found that both sofosbuvir alone and combination treatments effectively suppressed HCV replication.

In summary, these data suggest that combination treatment with a DAA plus LNA–anti-miR-122 can efficiently suppress HCV replication, possibly without the development of DAA-resistant mutants that tend to appear after treatment with single DAAs, especially simeprevir and daclatasvir. These data also suggest that treatment with 10 nM LNA–anti-miR-122 alone does not completely eliminate HCV from infected cells, although it does not seem to lead to the development of resistant mutants.

### Sequence Analysis

**Figure 3:**
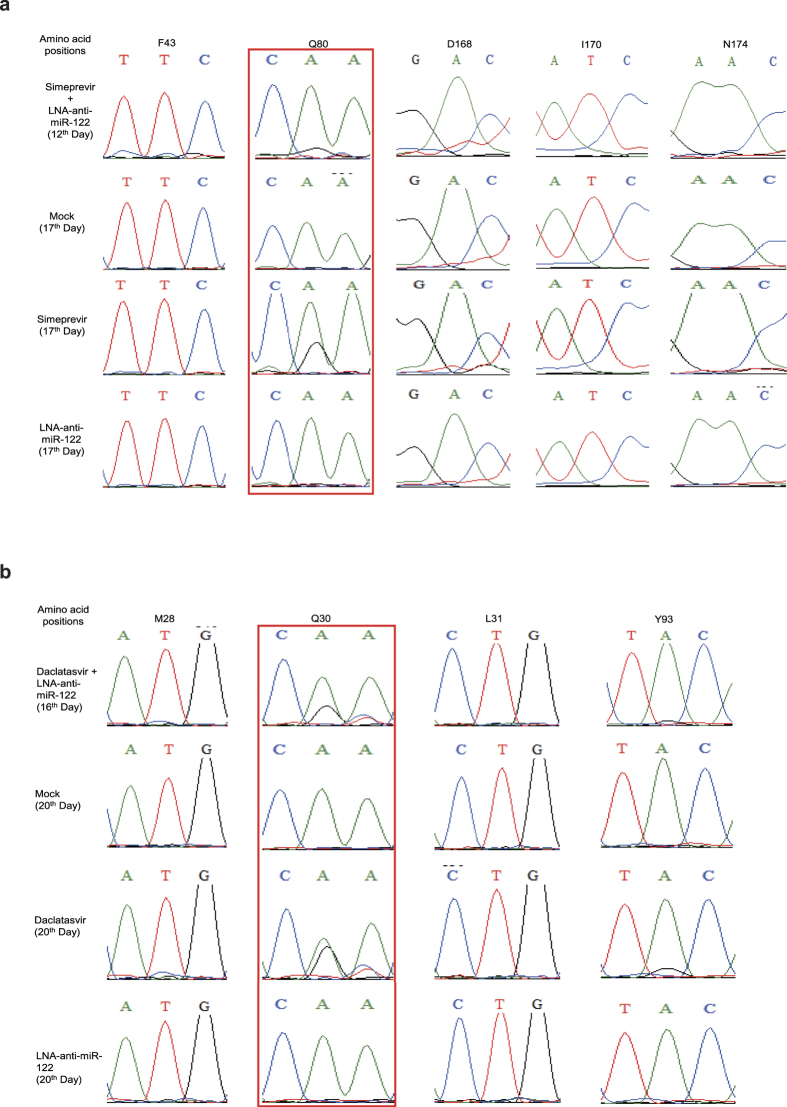
The results of population-based sequence analysis at the positions reported to be drug resistance sites. (**a**) In the simeprevir experiments, total RNA was extracted at every passage from the tat/2A-Neo-H77S-replicating cells. We performed analysis targeting the NS3 protease region on day 12 in the combination treatment group and on day 17 in the mock, LNA–anti-miR-122, and simeprevir single treatment groups. (**b**) In the daclatasvir experiments, total RNA was extracted from the tat/2A-Neo-replicating cells at every passage. We performed analysis targeting the N-terminal region of NS5A on day 16 in the combination treatment groups and day 20 in the mocks, LNA–anti-miR-122 and daclatasvir single treatment groups. The mutation site is shown within a red rectangular box.

Based on the above results, we hypothesized that combination treatments might suppress HCV replication effectively by preventing the emergence of DAA-resistant mutants. To confirm our hypothesis, we performed sequence analysis of HCV RNA in cells treated according to the conditions shown in Fig. 2a,c,e. First, we analyzed the HCV RNA from the cells replicating tat/2A-Neo-H77S and treated with either simeprevir or LNA–anti-miR-122. Population-based sequence analysis revealed that mutations at NS3 amino acid 80 were found in cells treated with simeprevir for 17 days and with simeprevir and LNA–anti-miR-122 for 12 days. The signal strength suggested that the mutations at amino acid 80 of NS3 were more dominant in the simeprevir single treatment group than in the combination treatment group. Significant simeprevir-resistance mutations were not observed in cells from the mock group or the LNA–anti-miR-122 single treatment group on day 17 (Fig. 3a).

We performed clonal sequence analysis to examine the HCV sequences in more depth. The F43S mutant was the most frequently observed in 52.4% of the clones analyzed on day 12 in the combination treatment group (Table 3). This mutant was also the most frequently observed in 75.0% of the clones analyzed at the same time point in the simeprevir single treatment group. Interestingly, it was difficult to identify the F43S mutation in the combination group via population-based analysis (Fig. 3a). In the mock group, the F43S mutant was much less common, and was found in 13.6% of the clones analyzed on day 12. We also observed a low frequency of Q80K and D168E mutants in the simeprevir single treatment group, and Q80R, D168E, and F43S + D168E mutants in the combination group on day 12. At the last time point, the Q80R, D168E, and N174K mutants were common, at 19.0%, 19.0%, and 23.8%, respectively, while F43S, Q80K, D168A, D168V, and I170T mutants were less frequently detected in the simeprevir single treatment group. Some highly drug-resistant mutants, such as D168A and D168V, were observed only in the simeprevir single treatment group.

In summary, the Q80R, D168E, and N174K mutants became more central over time in the simeprevir single treatment group (Table 3). We also analyzed the HCV RNA from the cells replicating H77S.3/Blast5A that were treated with either simeprevir or LNA–anti-miR-122 by population based and clonal sequence analysis. Interestingly, the patterns of the mutants and the way the mutants emerged differed from those seen with tat/2A-Neo-H77S (Supplementary Results 3, Supplementary Fig. S9 and Supplementary Table S3). However, these combination treatments could help to eliminate simeprevir-resistant mutants, which were more common in the simeprevir-single treatment in our study.

In a subsequent experiment, we analyzed the HCV RNA from cells replicating tat/2A-Neo-H77S treated with either daclatasvir or LNA–anti-miR-122. According to the results of population-based sequence analysis, the most frequently observed mutation was at amino acid Q30 in both the daclatasvir single and combination treatment groups. Conversely, there were no significant daclatasvir-resistance mutations in cells from the mock group or the LNA–anti-miR-122 single treatment group on day 20 (Fig. 3b).

We next performed clonal sequence analysis. The results showed that the Q30H and Q30R mutants were the most frequently observed and that some other mutants, such as M28T, L31M, and L31V, were less frequent in the daclatasvir single treatment group on day 11 (Table 4). In the combination treatment group, the Q30H and Q30R mutants were also the most frequent, and the M28T and L31M mutants less so on day 11. In the daclatasvir single treatment group, the Q30R and Q30H mutants were still the most frequently observed on days 16 and 20, while double mutants, such as Q30R + Y93H and Q30R + Y93R, were less frequent. Conversely, the M28T, Q30R, Q30H, and L31M mutants observed on day 11 were eliminated by day 16 in the combination treatment group.

We moved on to perform similar sequence analyses in the cells replicating tat/2A-Neo-H77S in the sofosbuvir single and combination treatment groups. Because the S282T mutation of NS5B is mainly characterized as a sofosbuvir-resistant mutation^26^,^27^, we performed population-based sequence analysis by focusing on the regions close to S282T. However, the S282T mutation was not observed in either group.

In summary, our study indicated that a DAA combined with LNA–anti-miR-122 has an enhanced resistance barrier compared with single DAA treatment.

## Discussion

In this study, we focused on a combination of miR-122 antagonism and DAA to overcome the drug resistance issues of DAA-based therapies. Previous studies have shown that miR-122 promotes HCV replication by improving translation, genome stability, and/or RNA amplification^15^. Therefore, sequestration of endogenous miR-122 by antisense oligonucleotides could suppress HCV replication. In fact, miR-122 antagonism has been proven to suppress HCV replication in HCV-infected cells, as well as chimpanzees and humans^12^,^16^,^17^. Several mutations that contribute to anti-miR-122 resistance have been identified in the regions close to two miR-122 binding sites, G28A and C3U^28^. In addition, the insertion of a host U3 snoRNA sequence into stem loop 1 of the HCV 5′ UTR impairs the response to miR-122 antagonism^18^. These mutations were identified in HCV cell culture systems, and have not been identified in studies of HCV-infected chimpanzees and humans. Therefore, anti-miR-122 therapy is considered to have a much higher barrier to the emergence of drug-resistant variants than DAAs.

We hypothesized that combination treatment with DAA and anti-miR-122 could effectively eliminate HCV from infected cells. This hypothesis was partially examined in a previous study by Ottosen *et al*.^22^. They showed that a combination of miravirsen with interferon-α2b and several DAAs displayed additive interactions and that miravirsen reduced the susceptibility of the DAA-resistant variants. However, the impacts of a combination of miravirsen and a DAA on HCV replication and the emergence of DAA-resistant mutants were not examined. Accordingly, in the present study, we investigated the usefulness of combination treatments in greater depth.

We found that the combination of LNA–anti-miR-122 and a DAA, such as simeprevir, daclatasvir, or sofosbuvir, reduced susceptibility to all DAAs in genotype 1a wild-type H77S.3/GLuc2A, although this depended on the combinations tested (Supplementary Fig. S4). The best combinations induced more dramatic changes in susceptibility to DAAs, as shown in Supplementary Fig. S4. In addition to wild-type HCV, combination treatment of DAA-resistant mutants with LNA–anti-miR-122 and DAA alleviated DAA resistance (Table 1; Supplementary Table S2 and Table 2), which is consistent with Ottosen’s study^22^. These results suggest that combination LNA–anti-miR-122 and DAA treatment should be effective in patients who have pre-existing RAVs.

A synergistic or additive antiviral effect was observed with LNA–anti-miR-122 and DAA treatment of DAA-resistant mutants, as well as wild-type virus, therefore we treated HCV-replicating cells with either LNA–anti-miR-122 or DAA for 2–4 weeks. We used full-length selectable HCV constructs (tat/2A-Neo-H77S and H77S.3/Blast5A) containing antibiotic-resistance genes. If the replication of these HCV RNAs was efficiently suppressed by either DAA or LNA–anti-miR-122 under antibiotic selection, the cells should die. On the other hand, if DAA-resistant mutants emerged, the cells would not die, despite increasing the DAA concentrations. When we treated the cells supporting HCV replication with only simeprevir or daclatasvir, HCV RNA levels quickly decreased, but then rebounded later, indicating that simeprevir-/daclatasvir-resistant mutants were established. While it was difficult to completely eliminate HCV by single treatment with LNA–anti-miR-122, HCV RNA levels were continuously suppressed without rebound during the course of treatment. This result indicates that the LNA–anti-miR-122–resistance virus is unlikely to emerge in our model. Importantly, when we treated HCV-replicating cells with LNA–anti-miR-122 at EC_50_ (10 nM) with gradually increasing concentrations of simeprevir or daclatasvir, HCV RNA levels sharply decreased, and all cells were finally killed by antibiotic selection. In the case of our experiments with sofosbuvir, all cells treated with LNA–anti-miR-122 plus sofosbuvir died and exhibited sharp decreases in HCV RNA levels. In contrast to the experiments with simeprevir and daclatasvir, not all cells treated with sofosbuvir died, even after sharp decreases in HCV RNA levels, and HCV continuously replicated at a very low level without evidence of rebound.

This phenomenon led us to further study the mechanisms of efficient suppression of HCV using only combination treatment. We hypothesized that the addition of LNA–anti-miR-122 to DAA would prevent the emergence of DAA-resistant mutants. To confirm this hypothesis, we performed two kinds of sequence analyses: population-based sequence analysis and clonal sequence analysis. Unexpectedly, we observed DAA-resistant variants in both the DAA single and combination treatment groups in the experiments with simeprevir and daclatasvir. However, the emergence of DAA-resistant mutants was more efficiently prevented in the combination group than the DAA single treatment group. We tested just 10 nM of LNA–anti-miR-122 in the present study. If we had used higher concentrations of LNA–anti-miR-122, DAA-resistant mutants could have been prevented more effectively. As significant DAA-resistance mutations were not observed in cells from the mock group or the LNA–anti-miR-122 single treatment group (Fig. 3a,b), LNA–anti-miR-122 itself does not have any effects on the emergence of DAA-resistant mutants. Thus, when LNA–anti-miR-122 was used with DAA, LNA–anti-miR-122 has the ability to partially prevent the emergence of DAA-resistant mutants which are easily selected or induced by a DAA single treatment.

In addition to its specific function in RNA replication, miR-122 could help to mask viral RNA from pathogen-recognition receptors, such as RIG-I (DDX58) and IFITs (interferon-induced protein with tetratricopeptide repeats)^29^,^30^. As these receptors sense RNAs with free 5′ triphosphates and are important antiviral factors, miR-122 could diminish innate antiviral defenses against HCV. In most of our experiments, we used Huh-7.5 cells, which lack interferon-mediated responses to HCV due to the absence of functional RIG-I and TLR3 (Toll-like receptor 3) expression^31^. Therefore, this sort of additional effect of miR-122 antagonism can be excluded. However, in humans, miR-122 antagonism could enhance an endogenous antiviral response, which could also help to prevent the development of DAA-resistant mutants.

In the experiments with sofosbuvir, we expected that single treatments would lead to the development of a S282T mutation in NS5B because this mutant is known to be quite unfit^11^, which is consistent with the low level of HCV RNA at a later time point. However, this mutation was not found in either the single treatment or combination treatment groups. In depth analyses of an entire NS5B region is required to find new mutations related to sofosbuvir resistance.

Clinical trials are required to confirm the safety and efficacy of miR-122 antagonists in humans. However, combination treatments with DAA and miR-122 antagonism are strongly expected to become options for patients who are unable to eliminate HCV via DAA regimens alone and/or for those who have developed several types of RAVs.

## Materials and Methods

### Reagents

Simeprevir and daclatasvir were purchased from Funakoshi Co., Ltd. (Tokyo, Japan) and sofosbuvir was obtained from Cayman Chemical (Ann Arbor, MI). Each was prepared in dimethyl sulfoxide (DMSO), the final concentration of which was 0.5%. LNA–anti-miR-122 (miravirsen or SPC3649) was synthesized by Nippon Gene Material Co., Ltd. (Toyama, Japan) and diluted in nuclease-free water.

### Cells

FT3-7 and Huh-7.5 (Huh-7 derivatives) cells were grown in Dulbecco’s modified Eagle’s medium supplemented with 10% fetal bovine serum, penicillin, streptomycin, L-glutamine, and non-essential amino acids.

### Plasmids

The plasmid pH77S.3^23^ is derived from pH77S^32^, an infectious molecular clone of genotype 1a HCV that contains an additional cell culture-adaptive mutation in E2 and a reversion of Q41R to Q41 in NS3. To easily monitor replication, the Gaussia Luciferase (GLuc) coding sequence, fused at its C terminus to the foot-and-mouth disease virus (FMDV) 2A autoprotease, was inserted between p7 and NS2 of pH77S.3 and designated pH77S.3/GLuc2A^23^. We used plasmids coding several NS3 mutants, which are described in our previous study^23^. NS3 mutations were inserted into pH77S.3/GLuc2A by applying the strategy used in our previous study^23^. The NS5A mutants in the pH77S.3/GLuc2A backbone, such as Q30R and L31V, were kindly provided by David R. McGivern and Stanley M. Lemon (The University of North Carolina at Chapel Hill)^25^.

The full-genomic selectable replicon harboring H77S, tat/2A-Neo-H77S, was modified from the corresponding subgenomic replicon^33^.

### RNA Transcription and Transfection

RNA was synthesized with the T7 RiboMAX™ Express Large Scale RNA Production System (Thermo Fisher Scientific Inc., Waltham, MA) after linearization of the plasmids with XbaI. Following treatment with RNase-free DNase to remove the template DNA, RNA was purified using the RNeasy Mini Kit (Qiagen, Hilden, Germany). RNA transfection was carried out using a TransIT mRNA Transfection Kit (Takara, Shiga, Japan), according to the manufacturer’s suggested protocol.

### LNA–anti-miR-122 Transfection

LNA–anti-miR-122 was transected into Huh-7.5 or FT3-7 cells using siPORT NeoFX Transfection Agent (Thermo Fisher Scientific Inc., Rockford, IL) according to the manufacturer’s instructions. Transfection mixtures were added to the cell culture plate and overlaid with cell suspensions. Cells were then incubated at 37 °C under 5% CO_2_. Three separate transfections were performed, and each was analyzed in triplicate.

### *In Vitro* Selection Experiments

HCV RNA (tat2A-Neo-H77S) was transfected into Huh-7.5 cells by electroporation as previously describeb^14^. Cells were then seeded in 10-cm dishes containing Dulbecco’s modified Eagle’s medium supplemented with 1 mg/mL G418. HCV-replicating cells were seeded in 10-cm dishes and transfected with LNA–anti-miR-122 at 10 nM using siPORT NeoFX Transfection Agent. DAAs (simeprevir, daclatasvir, or sofosbuvir) were added to the cells at EC_50_ values and their concentrations were gradually increased. LNA–anti-miR-122 was transfected into cells during twice-weekly passage, at which time total RNA was also extracted.

### Luciferase Activity Assay

Following RNA transfection, cell culture supernatant fluids were collected and fresh medium was added at 24-hour intervals. Secreted GLuc activity was measured in 50-μL aliquots of the supernatant using the GLuc Assay Kit (New England Biolabs, Ipswich, MA) according to the manufacturer’s suggested protocol. Luminescent signals were measured on a GloMax^®^-Multi + Microplate Multimode Reader with Instinct^®^ (Promega, Fitchburg, WI).

### Antiviral Activity Assays

Wild-type and mutant viral RNAs were transfected as described above. Medium containing serial dilutions of the antiviral compounds was freshly replaced at 24-hour intervals thereafter. Secreted GLuc activity was determined 72 hours post-transfection, as described above. The concentration of each compound required to reduce the amount of secreted GLuc activity by 50% (antiviral EC_50_) was determined using a three-parameter Hill equation (SigmaPlot 12.5, Systat Software, Inc. Chicago, IL). The synergy of the DAAs (simeprevir, daclatasvir, and sofosbuvir) plus LNA–anti-miR-122 for virus treatment was evaluated by the method of Chou and Talalay with CalcuSyn Software (CalcuSyn, The ComboSyn Inc. Paramus, NJ)^24^.

### qRT-PCR for HCV RNA

Total RNA was isolated using RNeasy Mini Kit (Qiagen, Hilden, Germany), and cDNA was synthesized with a high-capacity cDNA reverse transcription kit (Applied Biosystems, Carlsbad, CA). The primer pairs and probes for HCV and β-actin were obtained from the TaqMan assay reagents library. HCV RNA was detected as described previously^34^.

### Sequence Analysis of HCV RNAs

Total RNA was extracted from cells using the RNeasy Mini Kit (Qiagen) according to the instructions, and cDNA was synthesized with a high-capacity cDNA reverse transcription kit (Applied Biosystems) using random primers. For population-based sequence analysis of the NS3 protease (amino acids 1–180) and the N-terminal region of NS5A (amino acids 1–100), cDNA was amplified using the following set of primers: for NS3, NS3F1BamHI: 5′-TTAGGATCCGCGGCGTGTGG GGACATCAT-3′ containing an artificial BamHI site at its 5′ end and NS3R1XbaI: 5′-TTATCTAGAT TGCCGCTGCC AGTGGGAGC-3′ containing an artificial XbaI site at its 5′ end; for NS5A, NS5AF1: 5′-TCACTGCCAT ACTCAGCAG-3′ and NS5AR1: 5′-CAGTAGTCAT ACCCGATACG-3′. The DNA sequences of the PCR products were determined by the conventional Sanger method. In clonal sequence analysis, amplicons spanning the NS3 protease region were digested by BamHI and XbaI and then inserted into pGEM^®^-3Zf(+) vector (Promega), which was also digested with BamHI and XbaI. The amplicons spanning NS5A were directly inserted into pGEM^®^-T Easy Vector (Promega). We transformed DH5α Competent Cells (Takara) with the ligation products and then extracted plasmid DNA from the transformed DH5α cells. The DNA sequence of plasmid DNA was determined by the conventional Sanger method.

## Additional Information

**How to cite this article**: Liu, F. *et al*. Efficient Suppression of Hepatitis C Virus Replication by Combination Treatment with miR-122 Antagonism and Direct-acting Antivirals in Cell Culture Systems. *Sci. Rep.* **6**, 30939; doi: 10.1038/srep30939 (2016).

## Supplementary Material

Supplementary Information

## Figures and Tables

**Table 1:** EC_50_ Values (nM) of Simeprevir for NS3 Mutants in the Presence of Different Concentrations of LNA–anti-miR-122 (Also See Supplementary Table S1).

Mutants	Concentrations of LNA-anti-miR-122							
	0 nM	0.5 nM	1 nM	5 nM	8 nM	10 nM	20 nM	30 nM
WT	2.4	1.1	1.2	1.5	ND	1.6	2.0	1.8
Q80R	39.7	24.3	23.7	ND	ND	13.1	12.4	11.0
Q80K	34.2	23.0	26.3	19.2	16.7	18.8	23.8	17.9
R155K	189.1	149.7	94.4	93.8	190.4	140.3	139.1	173.1
R155T	64.9	14.2	30.3	29.9	16.9	ND	ND	ND
A156G	69.9	32.1	34.6	14.1	35.3	39.5	42.4	44.1
D168A	2688.6	1389.2	1058.6	1225.6	1463.3	973.5	ND	ND
D168H	2033.9	1378.0	1094.8	966.3	810.7	1045.6	ND	ND
D168T	1376.7	418.7	409.5	607.0	573.6	883.7	ND	ND
D168V	5566.7	3101.7	2808.2	ND	ND	1630.0	3623.1	5010.9
D168Y	923.0	884.5	884.3	429.7	248.7	880.3	ND	ND
Q80R + I170T	117.7	66.7	156.4	119.3	73.7	95.2	ND	ND
Q80R + D168E	1838.3	820.9	514.2	421.5	207.5	933.3	ND	ND
D168E + N174K	808.9	382.9	342.8	318.7	299.2	310.4	ND	ND

ND: not determined.

**Table 2:** EC_50_ Values (pM) of Daclatasvir for NS5A Mutants in the Presence of Different Concentrations of LNA–anti-miR-122.

Mutants	Concentrations of LNA-anti-miR-122					
	0 nM	0.5 nM	1 nM	5 nM	8 nM	10 nM
WT	18.8	13.2	17.0	15.4	ND	15.3
Q30R	4370.1	1133.4	975.1	696.4	1593.8	2230.9
L31V	9487.5	8250.2	3853.5	786.7	2332.2	5694.9

ND: not determined.

**Table 3:** Frequencies of NS3 Amino Acid Substitutions Determined by Clonal Sequence Analysis in tat/2A-Neo-H77S-replicating Cells Treated with Mock, Simeprevir, and Simeprevir plus LNA–anti-miR-122.

Mutants	Mock (day 12)	Simeprevir (day 12)	Simeprevir+ LNA-anti-miR-122 (day 12)	Mock (day 17)	Simeprevir (day 17)
F43S	3/22 (13.6%)	15/20 (75.0%)	11/21 (52.4%)	5/23 (21.7%)	2/21 (9.5%)
Q80R	0/22 (0%)	0/20 (0%)	1/21 (4.8%)	0/23 (0%)	4/21 (19.0%)
Q80K	0/22 (0%)	1/20 (5.0%)	0/21 (%)	0/23 (0%)	1/21 (4.8%)
D168E	0/22 (0%)	2/20 (10.0%)	1/21 (4.8%)	0/23 (0%)	4/21 (19.0%)
D168A	0/22 (0%)	0/20 (0%)	0/21 (0%)	0/23 (0%)	1/21 (4.8%)
D168V	0/22 (0%)	0/20 (0%)	0/21 (0%)	0/23 (0%)	1/21 (4.8%)
I170T	0/22 (0%)	0/20 (0%)	0/21 (0%)	0/23 (0%)	1/21 (4.8%)
N174K	0/22 (0%)	0/20 (0%)	0/21 (0%)	0/23 (0%)	5/21 (23.8%)
F43S + D168E	0/22 (0%)	0/20 (0%)	1/21 (4.8%)	0/23 (0%)	0/21 (0%)
WT	19/22 (86.4%)	2/20 (10.0%)	7/21 (33.3%)	18/23 (78.3%)	2/21 (9.5%)
Total	22	20	21	23	21

**Table 4:** Frequencies of NS5 Amino Acid Substitutions Determined by Clonal Sequence Analysis in tat/2A-Neo-H77S-replicating Cells Treated with Mock, Daclatasvir, and Daclatasvir plus LNA–anti-miR-122.

Mutants	Daclatasvir (day 11)	Daclatasvir+ LNA-anti-miR-122 (day 11)	Daclatasvir (day 16)	Mock (day 20)	Daclatasvir (day 20)
M28T	2/24 (8.3%)	1/24 (4.2%)	1/24 (4.2%)	0/24 (0%)	1/24 (4.2%)
Q30R	7/24 (29.2%)	5/24 (20.8%)	10/24 (41.7%)	0/24 (0%)	14/24 (58.3%)
Q30H	8/24 (33.3%)	9/24 (37.5%)	10/24 (41.7%)	0/24 (0%)	5/24 (20.8%)
Q30E	0/24 (0%)	0/24 (0%)	0/24 (0%)	0/24 (0%)	1/24 (4.2%)
L31M	1/24 (4.2%)	2/24 (8.3%)	0/24 (0%)	0/24 (0%)	0/24 (0%)
L31V	1/24 (4.2%)	0/24 (0%)	0/24 (0%)	0/24 (0%)	0/24 (0%)
Y93C	0/24 (0%)	0/24 (0%)	0/24 (0%)	0/24 (0%)	1/24 (4.2%)
Q30R + Y93H	0/24 (0%)	0/24 (0%)	1/24 (4.2%)	0/24 (0%)	0/24 (0%)
Q30R + Y93R	0/24 (0%)	0/24 (0%)	0/24 (0%)	0/24 (0%)	1/24 (4.2%)
WT	5/24 (20.8%)	7/24 (29.2%)	2/24 (8.3%)	24/24 (100.0%)	1/24 (4.2%)
Total	24	24	24	24	24
