# Supplementary material for: Efficient Suppression of Hepatitis C Virus Replication by Combination Treatment with miR-122 Antagonism and Direct-acting Antivirals in Cell Culture Systems
Source: Sci Rep. 2016 Aug 3;6:30939. doi: 10.1038/srep30939 (PMC4971519; doi:10.1038/srep30939)

Efficient Suppression of Hepatitis C Virus Replication by Combination Treatment with miR-122 Antagonism and Direct-acting Antivirals in Cell Culture Systems

Fanwei Liu[1], Tetsuro Shimakami[1,*], Kazuhisa Murai[1], Takayoshi Shirasaki[1], Masaya Funaki[1], Masao Honda[1], Seishi Murakami[1], Minkyung Yi[2], Hong Tang[3], and Shuichi Kaneko[1]

[1]Department of Gastroenterology, Kanazawa University Hospital, Kanazawa, Ishikawa 920-8641, Japan; [2]Department of Microbiology and Immunology, University of Texas Medical Branch at Galveston, Galveston, TX 77555-0144, USA; [3]Center of Infectious Diseases, West China Hospital, Sichuan University, Chengdu 610041, China

Supplementary Supporting Information Supplementary Results

S1. In Vitro Antiviral Activity of LNA–anti-miR-122 in Combination with DAAs

We investigated whether LNA–anti-miR-122 combined with simeprevir, daclatasvir, or sofosbuvir would show additive or synergistic antiviral activity. Briefly, we treated H77S.3/GLuc2A-replicating cells with various concentrations of LNA–anti-miR-122 and DAAs, and antiviral activity was evaluated by detecting GLuc activity. We calculated the combination index using the method of Chou and Talalay with CalcuSyn Software (Supplementary Table S1a-c)[1]. When the combination index was less than 0.8, the two treatments were considered to have a synergistic effect, while if it was between 0.8 and 1.2, the effect was considered additive. A combination of simeprevir and LNA–anti-miR-122 showed a mostly synergistic antiviral effect (Supplementary Fig. S4a). We obtained similar results with daclatasvir (Supplementary Fig. S4b) and sofosbuvir (Supplementary Fig. S4c). When we determined the EC_{50} values of simeprevir, daclatasvir, and sofosbuvir for wild-type virus in the presence of LNA–anti-miR-122, the EC_{50} was generally reduced for all of the DAAs tested, depending on the concentration of LNA–anti-miR-122 (Supplementary Fig. S4d-f). In conclusion, combination treatments with LNA–anti-miR-122 and DAAs showed synergistic or additive

antiviral effects on HCV genotype 1a H77S.

S2. In vitro selection of simeprevir in H77S.3/Blast5A-replicating cells.

We constructed a selectable and monocistronic full-genome repliconto confirm our results. For this purpose, we inserted a Blasticidin S-resistance coding gene into the NS5A region of H77S.3 in which GFP could be inserted[2], and Blasticidin S-resistance gene into H77S/AAG, in which the catalytic center of NS5B was changed from GDD to AAG. These HCV RNAs containing a Blasticidin S resistance coding gene within their NS5A regions, designated H77S.3/Blast5A and H77S/Blast5A/AAG, respectively, were transfected into Huh-7.5 cells. The transfected cells were selected with 10 μg/mL of Blascticidin S for 2 weeks. None of the cells transfected with H77S/Blast5A/AAG survived, while those transfected with H77S.3/Blast5A did. We detected the expression of HCV core in all of the Blasticidin S-resistant cells (Supplementary Fig. S5b), indicating that Blasticidin S resistance protein was functional even within the NS5A protein. Unfortunately, daclatasvir did not show any antiviral activity for H77S.3/Blast5A. We speculated that the Blasticidin S resistance protein would interfere with the interaction between daclatasvir and NS5A. Similar to daclatasvir, sofosbuvir did not show antiviral

activity in H77S.3/Blast5A.

Cells were maintained in the presence of 10 μg/mL Blasticidin S and split twice a week. LNA–anti-miR-122 (10 nM) was transfected at each split in the LNA–anti-miR-122 and LNA–anti-miR-122 plus DAA groups. Fresh DAA was also added at each split of the DAA and LNA–anti-miR-122 plus DAA groups. At each split, total RNA was extracted for the quantification of HCV RNA and sequence analysis.

We also performed a similar study using Huh-7.5 cells harboring H77S.3/Blast5A and treatment with either simeprevir or LNA–anti-miR-122. In the cells treated with only simeprevir, the amount of HCV RNA quickly decreased by day 2 (Supplementary Fig. S8a and S8b). However, it started to increase after day 8, and the cells survived even in the presence of 100 nM simeprevir, which strongly suggested that simeprevir-resistant mutants were appearing. In cells treated with 10 nM LNA–anti-miR-122 alone, the amount of HCV RNA gradually decreased until the last time point, suggesting that LNA–anti-miR-122–resistant mutants were unlikely to occur. In contrast, the amount of HCV RNA sharply decreased in the cells treated with simeprevir plus LNA–anti-miR-122, an event that was also observed in cells treated with only simeprevir. Although we continuously detected small amounts of HCV RNA

after day 2, all cells were dead by day 16. The results obtained from cells harboring tat/2A-Neo-H77S and those harboring H77S.3/Blast5A were quite similar.

S3. Sequence Analysis

Next, we analyzed the HCV RNA from the cells replicating H77S.3/Blast5A treated with either simeprevir or LNA–anti-miR-122. Interestingly, the pattern of the mutants and the way mutants emerged were different from those seen with tat/2A-Neo-H77S. Population-based sequence analysis revealed that the R155K mutation was the most dominant mutation in both the simeprevir single and combination treatment groups on days 20 and 16, respectively. Significant simeprevir-resistance mutations were not observed in the cells from the mock group or the LNA–anti-miR-122 single treatment group on day 20 (Supplementary Fig. S9).

We performed clonal sequence analysis to investigate the HCV sequences further. As shown in Supplementary Table 3, D168E and R155K mutants were observed frequently in 21.7% and 17.4%, of the clones analyzed on day 12 in the simeprevir single treatment group, respectively. We also observed the

following mutants less frequently on day 12 in this group: F43S/L, Q80R, S122R, I170T, N174K, Q80R+I170T, and R155K+D168E. Similar to the simeprevir single treatment group, R155K mutant was also the most frequently observed in combination treatment groups. However, the mutants at the 43th, 168th and 170th positions were observed less frequently and the mutants at the 156th position more frequently compared with the simeprevir single treatment group on day 12. In both groups, the Q80R and N174K mutants, both of which were observed frequently in the experiments involving tat/2A-Neo-H77S, were observed less often. In addition to single mutants, we found some double mutants such as Q80R+I170T and R155K+D168E on day 12, Q80R+R155K and A156V+D168E on day 16, and F43L+D168E and Q80R+D168A on day 20 in the simeprevir single treatment group. On the other hand, only one double mutant, F43S+R155K, was observed in the combination treatment group. The highly drug-resistant mutant D168V was observed in the simeprevir single treatment group only.

The frequency of the R155K mutant continuously increased in the simeprevir single treatment group over time, becoming detectable in 17.4%, 41.7%, and 75.0% of the clones analyzed on days 12, 16, and 20, respectively. Conversely,

the R155K mutant sharply decreased in the combination group, from 28.6% to 5.0% in the clones analyzed on days 12 and 16, respectively. We noticed that the predominant mutant in the simeprevir single treatment group was R155K, although several kinds of mutants, such as F43S, Q80R, R155K, A156T/G, D168E, and F43S+R155K, were observed at low frequency in the combination group. These results showed that combination treatment could help to eliminate simeprevir-resistant mutants, even after the emergence of simeprevir-resistant mutants, such as R155K, in a small population.

Supplementary Materials and Methods

Plasmids

The plasmid pH77S.3 [3] is derived from pH77S [4], an infectious molecular clone of genotype 1a HCV that contains an additional cell culture-adaptive mutation in E2 and a reversion of Q41R to Q41 in NS3. To insert the Blasticidin S coding gene into NS5A between the amino acids at positions 387 and 388, MluI and EcoRV restriction sites were created by site-directed mutagenesis. Then, DNA coding Blasticidin S, amplified by primers containing MluI and EcoRV restriction sites, was inserted into pH77S.3/GLuc2A to create H77S.3/Blast5A.

RNA Oligonucleotides

A negative control of LNA-modified single-stranded RNA oligonucleotides, negative control A, was purchased from EXIQON (Vedbaek, Denmark). The sequence of negative control A was 5′-TAACACGTCTATACGCCCA-3′. FAM-labeled 2′-*O*-methyl (2′OMe) oligonucleotides anti-miR-122 was synthesized by Nippon Gene Material Co., Ltd. (Toyama, Japan). The sequence of ant-miR-122 is 5′-AGACACAAACACCAUUGUCACACUCCACAGC-3′[5] and FAM was added to the 5′ end.

MTT Assay

The cells were seeded into 96-well plates and transfected with LNA-anti-miR-122 using siPORT NeoFX Transfection Agent according to the manufacturer’s suggested protocol. We added 10 μL of Cell Counting Kit-8 (CCK-8) (Dojindo Molecular Technologies, Inc., Rockville, USA) solution to each well of the plate 48 h later. The plates were incubated for 4 h at 37 °C, and the absorbance was measured at 450 nm using a microplate reader (Sunrise, Männedorf, Switzerland).

In vitro Selection Experiments

HCV RNA (H77S.3/Blast5A) was transfected into Huh-7.5 cells by electroporation as we previously showed[6]. Cells were then seeded in 10-cm dishes containing Dulbecco's modified Eagle's medium supplemented with 10 μg/mL Blasticidin S. HCV-replicating cells were seeded in 10-cm dishes and transfected with LNA–anti-miR-122 at 10 nM using siPORT NeoFX Transfection Agent. Simeprevir was added to the cells at their EC_{50} values with a gradual increase in their concentrations. LNA–anti-miR-122 was transfected into cells during the twice-weekly passage, and total RNA was extracted at the same time.

Immunostaining

For immunofluorescence staining, cells were washed twice with PBS and fixed in 4% paraformaldehyde for 15 min at room temperature. After washing again with PBS, cells were permeabilized with 0.05% Triton X-100 in PBS for 15 min at room temperature. Cells were incubated in a blocking solution (3% bovine serum albumin in PBS) for 30 min and then with anti-core protein monoclonal antibodies (Thermo Fisher Scientific Inc., Rockford, IL). The fluorescent secondary antibodies were Alexa Fluor 488-conjugated anti-mouse IgG antibodies (Thermo Fisher Scientific Inc. Rockford, IL). Nuclei were labeled with DAPI. Imaging was performed on a BIOREVO fluorescence microscope

(Keyence Corporation, Osaka, Japan).

qRT-PCR for HCV RNA

Total RNA was isolated using an RNeasy Mini Kit (Qiagen, Hilden, Germany), and cDNA was synthesized with a high-capacity cDNA reverse transcription kit (Applied Biosystems, Carlsbad, CA). The primer pairs and probes for HCV and β-actin were obtained from the TaqMan assay reagents library. HCV RNA was detected as described previously[7]. HCV copies numbers were calculated following the previous method[8].

References for Supplementary Supporting Information

1 Chou , T. C. & Talalay, P. Analysis of combined drug effects: a new look at a very old problem. *Trends in Pharmacological Sciences* **4**, 450-454, doi:10.1016/0165-6147(83)90490-X (1983).

2 Svarovskaia, E. S. *et al.* Infrequent development of resistance in genotype 1-6 hepatitis C virus-infected subjects treated with sofosbuvir in phase 2 and 3 clinical trials. *Clinical infectious diseases : an official publication of the Infectious Diseases Society of America* **59**, 1666-1674, doi:10.1093/cid/ciu697 (2014).

3 Shimakami, T. *et al.* Protease inhibitor-resistant hepatitis C virus mutants with reduced fitness from impaired production of infectious virus. *Gastroenterology* **140**, 667-675, doi:10.1053/j.gastro.2010.10.056 (2011).

4 Yi, M., Villanueva, R. A., Thomas, D. L., Wakita, T. & Lemon, S. M. Production of infectious genotype 1a hepatitis C virus (Hutchinson strain) in cultured human hepatoma cells. *Proc Natl Acad Sci U S A* **103**, 2310-2315, doi:10.1073/pnas.0510727103 (2006).

5 Jopling, C. L., Yi, M., Lancaster, A. M., Lemon, S. M. & Sarnow, P. Modulation of hepatitis C virus RNA abundance by a liver-specific

MicroRNA. *Science* **309**, 1577-1581, doi:10.1126/science.1113329 (2005).

6 Shimakami, T. *et al.* Stabilization of hepatitis C virus RNA by an Ago2-miR-122 complex. *Proc Natl Acad Sci U S A* **109**, 941-946, doi:10.1073/pnas.1112263109 (2012).

7 Honda, M., Shimazaki, T. & Kaneko, S. La protein is a potent regulator of replication of hepatitis C virus in patients with chronic hepatitis C through internal ribosomal entry site-directed translation. *Gastroenterology* **128**, 449-462 (2005).

8 Shirasaki, T. *et al.* MicroRNA-27a regulates lipid metabolism and inhibits hepatitis C virus replication in human hepatoma cells. *J Virol* **87**, 5270-5286, doi:10.1128/JVI.03022-12 (2013).

Supplementary Figure Legends

Supplementary Fig. S1. Evaluation of anti-miR-122 transfection efficiency.

Huh-7.5 cells were transfected with 10 nM of FAM-labeled anti-miR-122 2′OMe using siPORT NeoFX Transfection Agent. Transfected cells were fixed and stained with DAPI 6 h later. The signal from FAM-labeled anti-miR-122 2′OMe was detected by fluorescence microscopy. The color blue represents DAPI and the green dot represents FAM-labeled anti-miR-122 2′OMe.

Supplementary Fig. S2. CC_{50} of LNA-anti-miR-122 in different cell lines.

Huh-7.5 cells, FT3-7 cells, and Huh-7.5 cells harboring tat/2A-Neo-H77S replicon were seeded into 96-well plates and transfected with LNA-anti-miR-122 in serial dilutions using siPORT NeoFX Transfection Agent.

CC_{50} levels were determined based on absorbance from MTT assays, and all results were normalized to the absorbance in the mock group. The CC_{50} of LNA-anti-miR-122 in Huh-7.5 cells is shown in Fig S2a, in FT3-7 cells in Fig S2b, and in Huh-7.5 cells harboring tat/2A-Neo-H77S replicon in Fig S2c. Results are shown as the means + SEM.

Supplementary Fig. S3. The effect of LNA-modified single-stranded RNA (LNA-RNA-Control) on HCV replication. FT3-7 cells were transfected with H77S.3/GLuc2A RNA, H77S.3/GLuc2A L31V(NS5A) RNA or H77S.3/GLuc2A D168A(NS3) RNA, transfected with mock, 10 nM LNA-anti-miR-122, or LNA-RNA-Control 24 h later. Medium was replaced at 24-h intervals. Secreted GLuc activity was determined at 72 h post-transfection. CLuc activity was normalized to that of the mock in each HCV RNA set to 100 %. Results are shown as the means + SEM.

Supplementary Fig. S4. The combination index between LNA–anti-miR-122 and DAAs. The combination index between LNA–anti-miR-122 and each DAA—simeprevir (a), daclatasvir (b), and sofosbuvir (c)—was calculated. Results are shown as the means + SD. A combination index value between 0.8 and 1.2 represents an additive effect, whereas a value less than

0.8 indicates synergy. In addition, we also analyzed the EC_{50} values of simeprevir (d), daclatasvir (e), and sofosbuvir (f) in the presence of different amounts of LNA–anti-miR-122. We transfected H77S.3/GLu2A RNA into FT3-7 cells transfected them with a different amount of LNA–anti-miR-122 24 h later. At the same time, simeprevir, daclatasvir, or sofosbuvir was added in a serial dilution. We replaced the medium with fresh medium containing DAA 48 h later. After 24 h, the supernatant was harvested, and GLuc activity was determined. The EC_{50} of simeprevir (d), daclatasvir (e), and sofosbuvir (f) in the presence of different concentrations of LNA–anti-miR-122 was determined from GLuc activity. Results are shown as the means + SD.

Supplementary Fig. S5. Establishment of HCV replicating cell lines (a) Establishment of the tat/2A-Neo-H77S-replicating cell line. We transfected tat/2A-Neo-H77S RNA into Huh-7.5 cells by electroporation. The transfected cells were then seeded in a 10-cm dish and incubated in the presence of 1 mg/mL G418. Two weeks later, the HCV core protein was detected by an immunofluorescence assay. Blue represents DAPI, and green represents the HCV core protein. (b) Establishment of the H77S.3/Blast5A-replicating cell line. We transfected H77S.3/Blast5A RNA into Huh-7.5 cells by electroporation. The

transfected cells were then seeded in a 10-cm dish and incubated in the presence of 10 μg/mL Blasticidin S. Two weeks later, the HCV core protein was detected by an immunofluorescence assay. Blue color represents DAPI, and green represents the HCV core protein.

Supplementary Fig. S6. Effective suppression of HCV replication by LNA–anti-miR-122 in HCV RNA-transfected cells. We transfected HJ3-5/GLuc2A RNA into Huh-7.5 cells using TransIT®-mRNA Transfection Kit. After 24 h, LNA–anti-miR-122, LNA-RNA-control, or mock was transfected into cells using siPORT™ NeoFX™ Transfection Agent. The final concentration of LNA–anti-miR-122 or LNA-control was 10 nM. We collected the medium and replaced it with fresh medium every 24 h. The GLuc activity of the medium was determined. Results were normalized to that of the no treatment group. The results are shown as the means +SEM.

Supplementary Fig. S7. Evaluation of HCV RNA copy numbers from in vitro selection experiments. Huh-7.5 cells harboring tat/2A-Neo-H77S or H77S.3/Blast5A were maintained in the presence of 1 mg/mL of G418 or 10 μg/mL of Blasticidin S. HCV copy numbers were calculated for in vitro selection experiments of simeprevir in huh-7.5 cells harboring tat/2A-Neo-H77S (a), in

vitro selection experiments of simeprevir in huh-7.5 cells harboring H77S.3/Blast5A (b), in vitro selection experiment of daclatasvir in huh-7.5 cells harboring tat/2A-Neo-H77S (c), and in vitro selection of sofosbuvir in Huh-7.5 cells harboring tat/2A-Neo-H77S(d). The arrows in the upper part of the figure represent the points in time when LNA-anti-miR-122 was transfected and DAAs added. The results are shown as the means + SEM.

Supplementary Fig. S8. In vitro selection experiments in H77S.3/Blast5A-replication cells with simeprevir. Huh-7.5 cells harboring H77S.3/Blast5A were treated with mock, simeprevir, LNA-anti-miR-122 or simeprevir plus LNA–anti-miR-122 in the presence of 10 μg/mL Blasticidin S. The concentration of simeprevir was gradually increased and LNA–anti-miR-122 was transfected into Huh-7.5 cells twice a week during cell passage. At the same time, total RNA was extracted for quantitation and sequence analysis. (a) A schematic representation of the simeprevir experiments in H77S.3/Blast5A-replicating cells. (b) The quantity of HCV RNA was normalized to that of β-actin mRNA in each group. The arrows in the upper part of the figure represent the points in time when LNA-anti-miR-122 was transfected and simeprevir added. The results are shown as the means +

SEM.

Supplementary Fig. S9. The results of population-based sequence analysis at the positions reported to be drug resistance sites. In the simeprevir experiments, total RNA was extracted at every passage from the H77S.3/Blast5A-replicating cells. We performed analysis targeting the NS3 protease region on day 16 in the combination treatment group and on day 20 in the control, LNA–anti-miR-122 and simeprevir single treatment groups. The mutation site is highlighted by a red rectangular box.

Supplementary Tables

Simeprevir (nM)	LNA-anti-miR-122 (nM)	GLuc activity (LU/mL)	Fa	CI
0.5 (0.25×EC_{50})	1.5 (0.15×EC_{50})	414457	0.406611	0.41
1 (0.5×EC_{50})	3 (0.3×EC_{50})	326809.25	0.494818	0.552
2 (1.0×EC_{50})	6 (0.6×EC_{50})	178218.75	0.714151	0.443
4 (2.0×EC_{50})	12 (1.2×EC_{50})	40695.25	0.932274	0.225
8 (4.0×EC_{50})	24 (2.4×EC_{50})	6410	0.988314	0.122

Supplementary Table S1a

Daclatasvir (pM)	LNA-anti-miR-122 (pM)	GLuc activity (LU/mL)	Fa	CI
1 (0.1×EC_{50})	100 (0.01×EC_{50})	1212890	0.745502	0.042
5 (0.5×EC_{50})	500 (0.05×EC_{50})	1001870	0.752072	0.203
10 (1.0×EC_{50})	1000 (0.1×EC_{50})	843782	0.875291	0.214
20 (2.0×EC_{50})	2000 (0.2×EC_{50})	593548	0.913534	0.314
100 (10×EC_{50})	10000 (1.0×EC_{50})	325404	0.954515	0.938

Supplementary Table S1b

Sofosbuvir (pM)	LNA-anti-miR-122 (pM)	GLuc activity (LU/mL)	Fa	CI
10000 (0.2×EC_{50})	100 (0.01×EC_{50})	254707	0.530729	0.462
50000 (1.0×EC_{50})	500(0.05×EC_{50})	146406	0.747337	0.537
100000 (2.0×EC_{50})	1000 (0.1×EC_{50})	50492	0.915437	0.46
200000 (4.0×EC_{50})	2000 (0.2×EC_{50})	19223	0.961887	0.53
300000 (6.0×EC_{50})	3000 (0.3×EC_{50})	11018	0.977131	0.564

Supplementary Table S1c

Supplementary Table S1. Evaluation of the Combination Index between DAAs and LNA–anti-miR-122 by the Method of Chou and Talalay with CalcuSyn Software

Fa indicates the fraction affected by the dose, and CI indicates the combination index.

We transfected H77S.3/GLuc2A RNA into FT3-7 cells using a *Trans*IT®-mRNA Transfection Kit and transfected LNA–anti-miR-122 24 h later at different concentrations using siPORT™ *NeoFX*™ Transfection Agent. DAAs were added into the medium at different concentrations. We replaced the medium with fresh medium containing DAA at the same serial dilutions 48h later. The supernatant was harvested from the cultures 24 h later and GLuc activity was determined. Finally, the combination index (CI) and Fa of simeprevir (a), daclatasvir (b), and sofosbuvir (c) were analyzed from the GLuc activity using the method of Chou and Talalay with CalcuSyn Software.

Mutants	Concentrations of LNA-anti-miR-122							
	0 nM	0.5 nM	1 nM	5 nM	8 nM	10 nM	20 nM	30 nM
V36G	14.9	13.4	10.9	ND	ND	10.2	6.0	10.1
F43S	47.7	39.3	21.0	ND	ND	ND	ND	ND
S122R	137.8	106.1	95.9	ND	96.5	126.7	ND	ND
D168G	43.4	22.0	36.1	ND	ND	18.3	ND	ND
D168E	87.0	54.3	75.8	63.5	86.2	90.0	ND	ND
D168N	23.8	17.0	17.3	ND	ND	10.5	9.9	14.5
I170T	25.4	18.0	19.2	28.6	24.4	16.6	16.1	15.0
N174K	12.3	5.8	10.2	ND	ND	8.9	6.7	6.8
I132V+D168E	148.7	119.7	78.5	102.7	142.6	149.8	ND	ND

Supplementary Table S2. EC_{50} Values of Simeprevir for NS3 Mutants in the Presence of Different Concentrations of LNA–anti-miR-122

Mutants	Simeprevir (day 12)	Simeprevir+ LNA-anti-miR-122 (day 12)	Simeprevir (day 16)	Simeprevir+ LNA-anti-miR-122 (day 16)	Mock (day 20)	Simeprevir (day 20)
F43S	3/23(13.0%)	1/21 (4.8%)	0/24 (0%)	3/20(15.0%)	0/24 (0%)	0/24 (0%)
F43L	1/23 (4.3%)	0/21 (0%)	0/24 (0%)	0/20 (0%)	0/24 (0%)	0/24 (0%)
Q80R	1/23 (4.3%)	0/21 (0%)	0/24 (0%)	4/20(20.0%)	0/24 (0%)	1/24 (4.2%)
S122R	1/23 (4.3%)	0/21 (0%)	0/24 (0%)	0/20 (0%)	0/24 (0%)	0/24 (0%)
R155K	4/23(17.4%)	6/21(28.6%)	10/24(41.7%)	1/20 (5.0%)	0/24 (0%)	18/24(75.0%)
R155S	0/23 (0%)	1/21 (4.8%)	0/24 (0%)	0/20 (0%)	0/24 (0%)	0/24 (0%)
R155T	0/23 (0%)	2/21 (9.5%)	0/24 (0%)	0/20 (0%)	0/24 (0%)	0/24 (0%)
A156T	0/23 (0%)	3/21(14.3%)	2/24 (8.3%)	3/20(15.0%)	0/24 (0%)	1/24 (4.2%)
A156G	0/23 (0%)	2/21 (9.5%)	1/24 (4.2%)	1/20 (5.0%)	0/24 (0%)	1/24 (4.2%)
A156V	0/23 (0%)	0/21 (0%)	1/24 (4.2%)	0/20 (0%)	0/24 (0%)	0/24 (0%)
D168E	5/23(21.7%)	1/21 (4.8%)	3/24(12.5%)	1/20 (5.0%)	0/24 (0%)	1/24 (4.2%)
D168V	0/23 (0%)	0/21 (0%)	1/24 (4.2%)	0/20 (0%)	0/24 (0%)	0/24 (0%)
I170T	2/23 (8.7%)	1/21 (4.8%)	1/24 (4.2%)	0/20 (0%)	1/24 (4.2%)	0/24 (0%)
N174K	1/23 (4.3%)	0/21 (0%)	0/24 (0%)	0/20 (0%)	0/24 (0%)	0/24 (0%)
F43S+R155K	0/23 (0%)	0/21 (0%)	0/24 (0%)	1/20 (5.0%)	0/24 (0%)	0/24 (0%)
F43L+D168E	0/23 (0%)	0/21 (0%)	0/24 (0%)	0/20 (0%)	0/24 (0%)	1/24 (4.2%)
Q80R+R155K	0/23 (0%)	0/21 (0%)	1/24 (4.2%)	0/20 (0%)	0/24 (0%)	0/24 (0%)
Q80R+D168A	0/23 (0%)	0/21 (0%)	0/24 (0%)	0/20 (0%)	0/24 (0%)	1/24 (4.2%)
Q80R+I170T	1/23 (4.3%)	0/21 (0%)	0/24 (0%)	0/20 (0%)	0/24 (0%)	0/24 (0%)
R155K+D168E	1/23 (4.3%)	0/21 (0%)	0/24 (0%)	0/20 (0%)	0/24 (0%)	0/24 (0%)
A156V+D168E	0/23 (0%)	0/21 (0%)	1/24 (4.2%)	0/20 (0%)	0/24 (0%)	0/24 (0%)
WT	3/23(13.0%)	4/21(19.0%)	3/24(12.5%)	6/20(30.0%)	23/24(95.8%)	0/24 (0%)
Total	23	21	24	20	24	24

Supplementary Table S3. Frequencies of NS3 Amino Acid Substitutions Determined by Clonal Sequence Analysis in H77S.3/Blast5A-replicating Cells Treated with Mock, Simeprevir, and Simeprevir plus LNA–anti-miR-122 Treatments

Supplementary Fig.S1

Mock

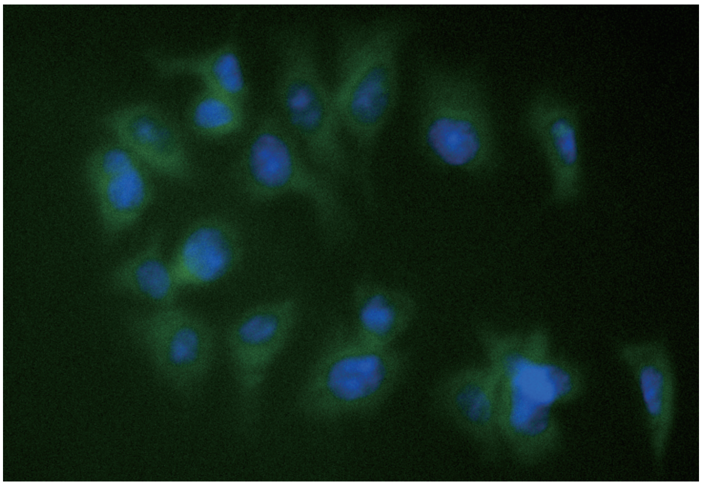

FAM-labeled anti-miR-122 2’OMe Transfected

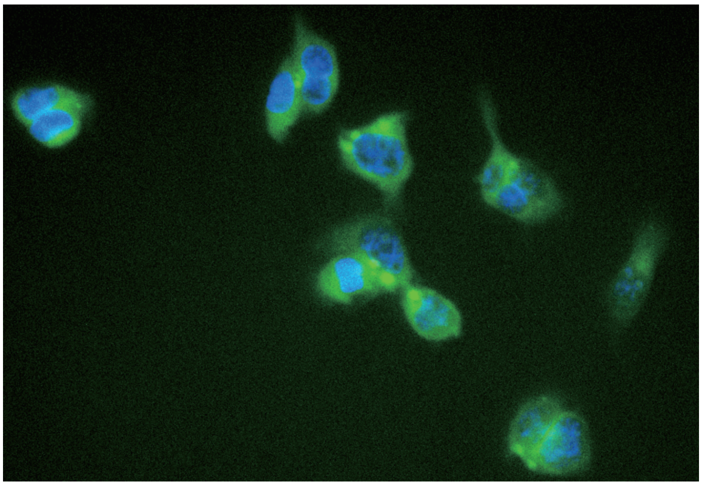

Blue; DAPI, Green dot; FAM-labeled anti-miR-122 2’OMe

Supplementary Fig.S2

a

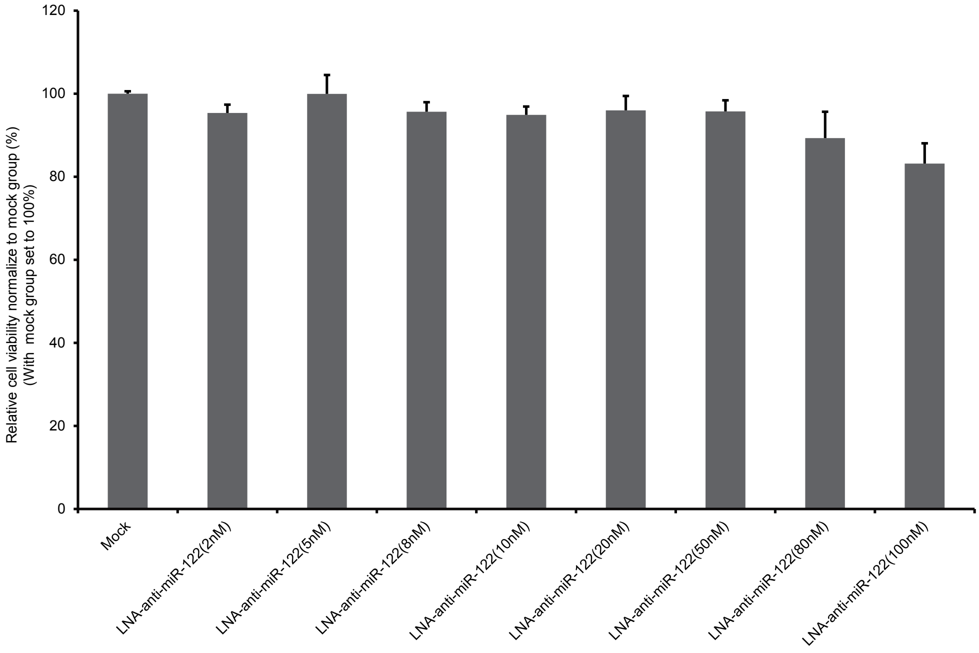

b

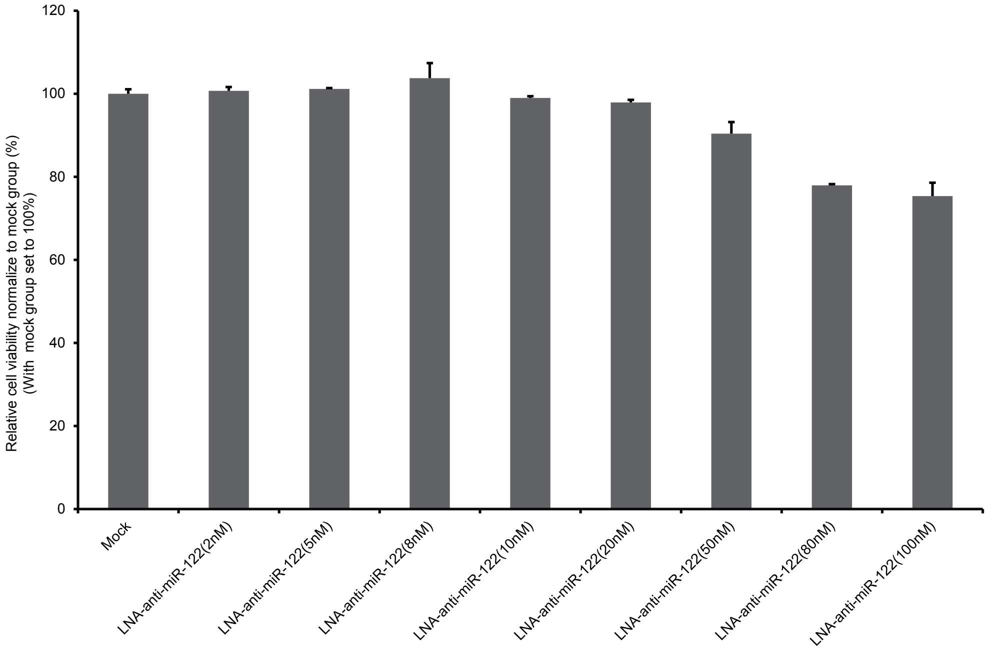

c

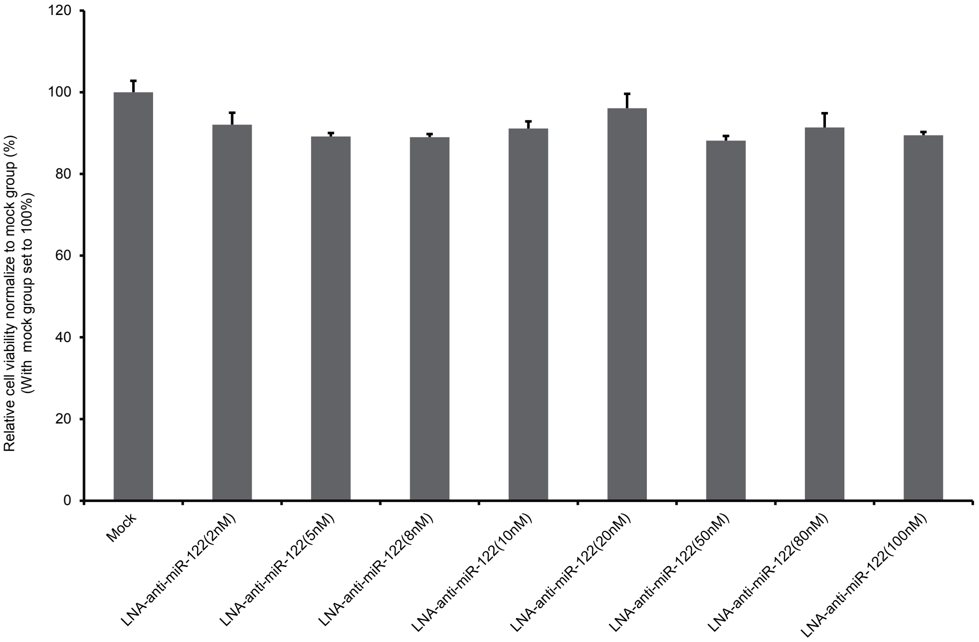

Supplementary Fig.S3

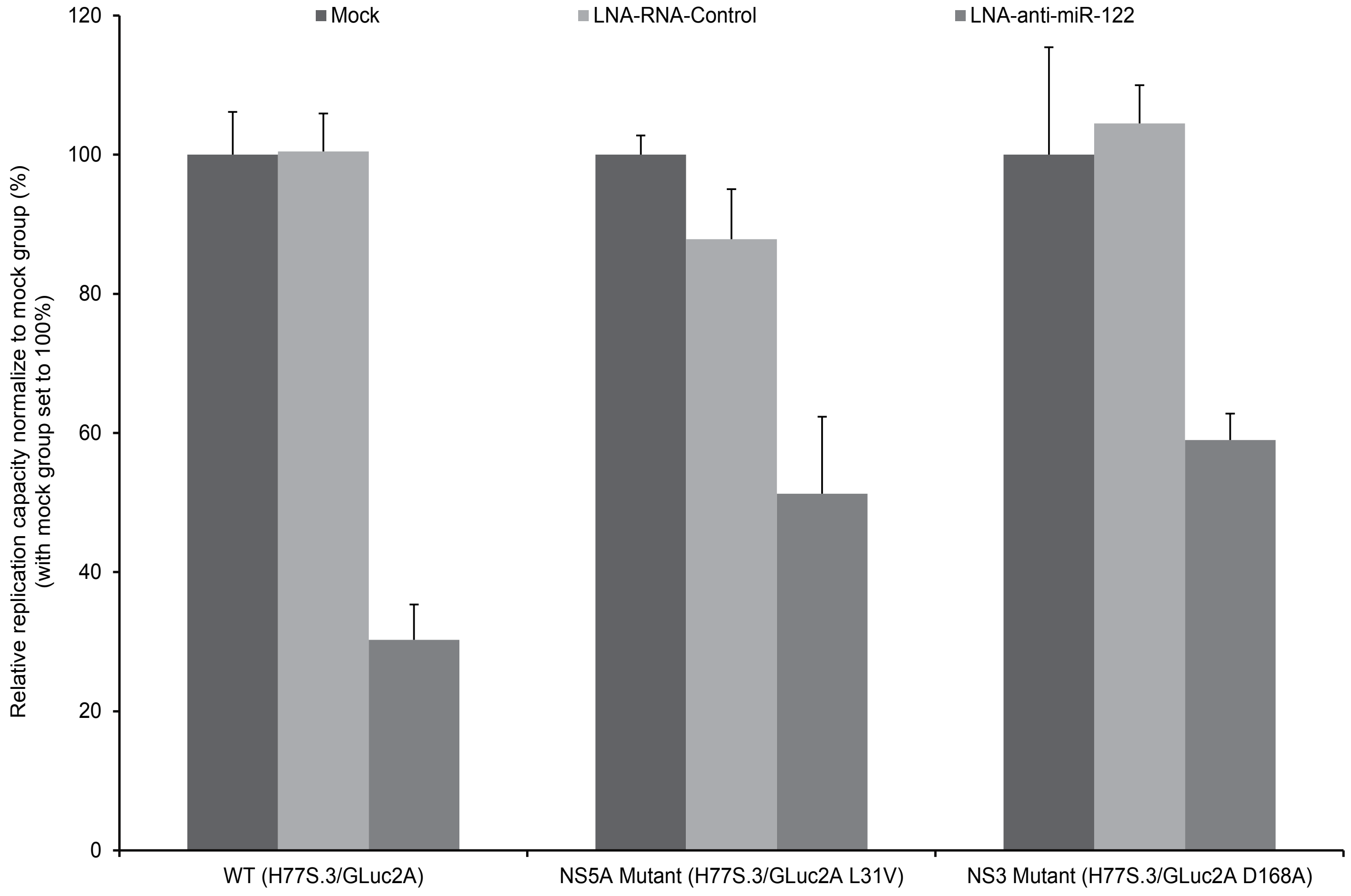

Supplementary Fig.S4

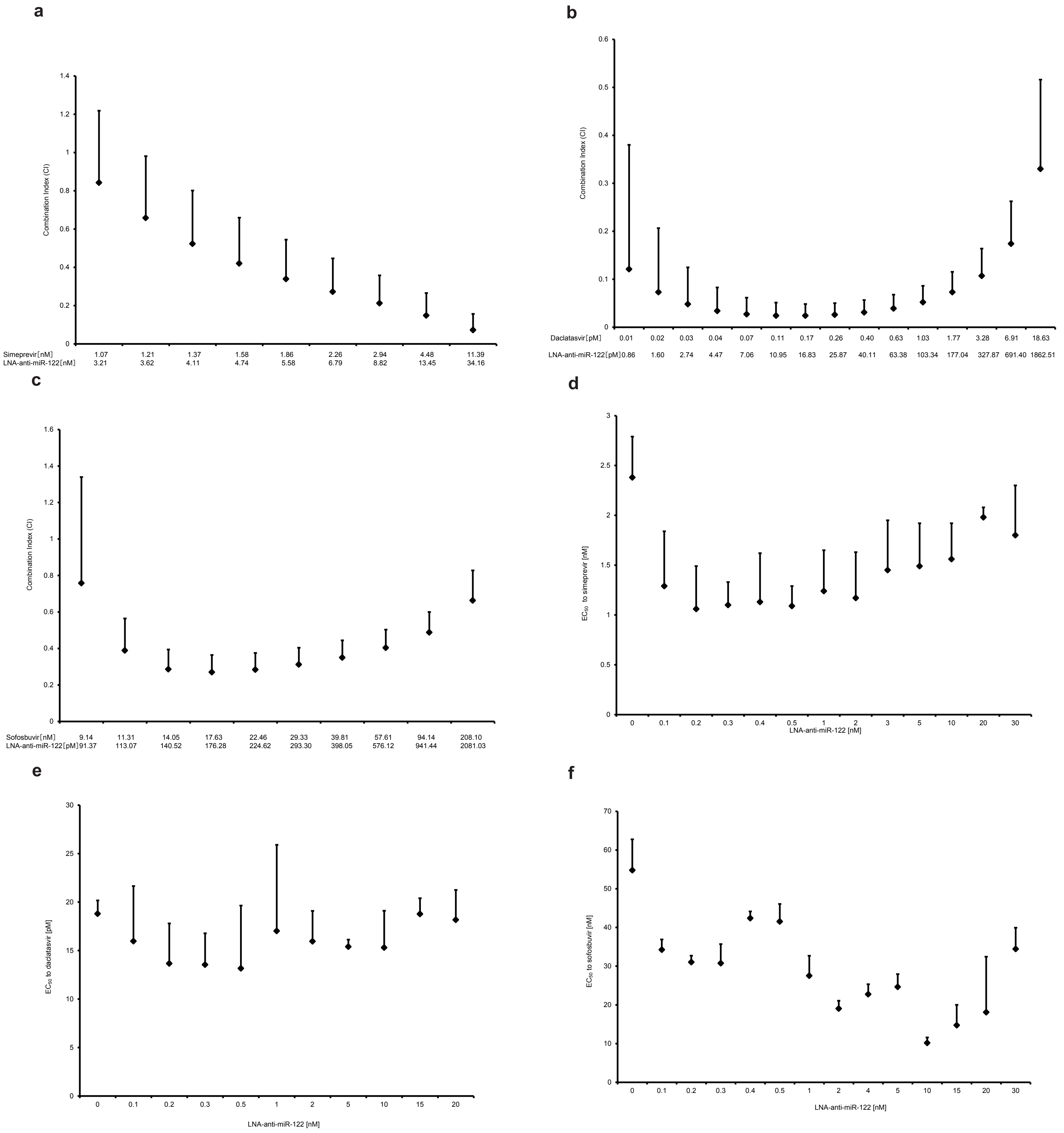

Supplementary Fig.S5

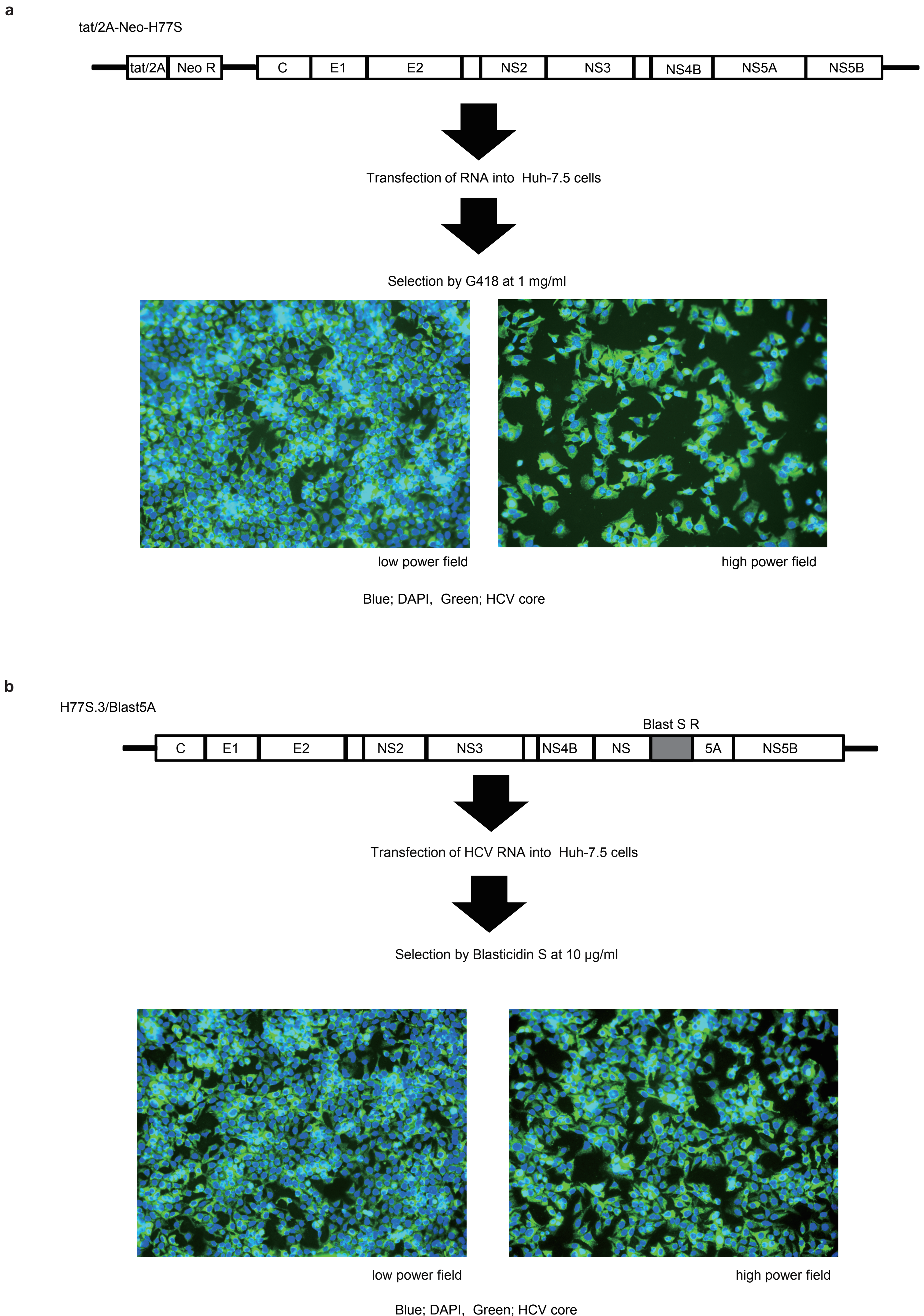

Supplementary Fig.S6

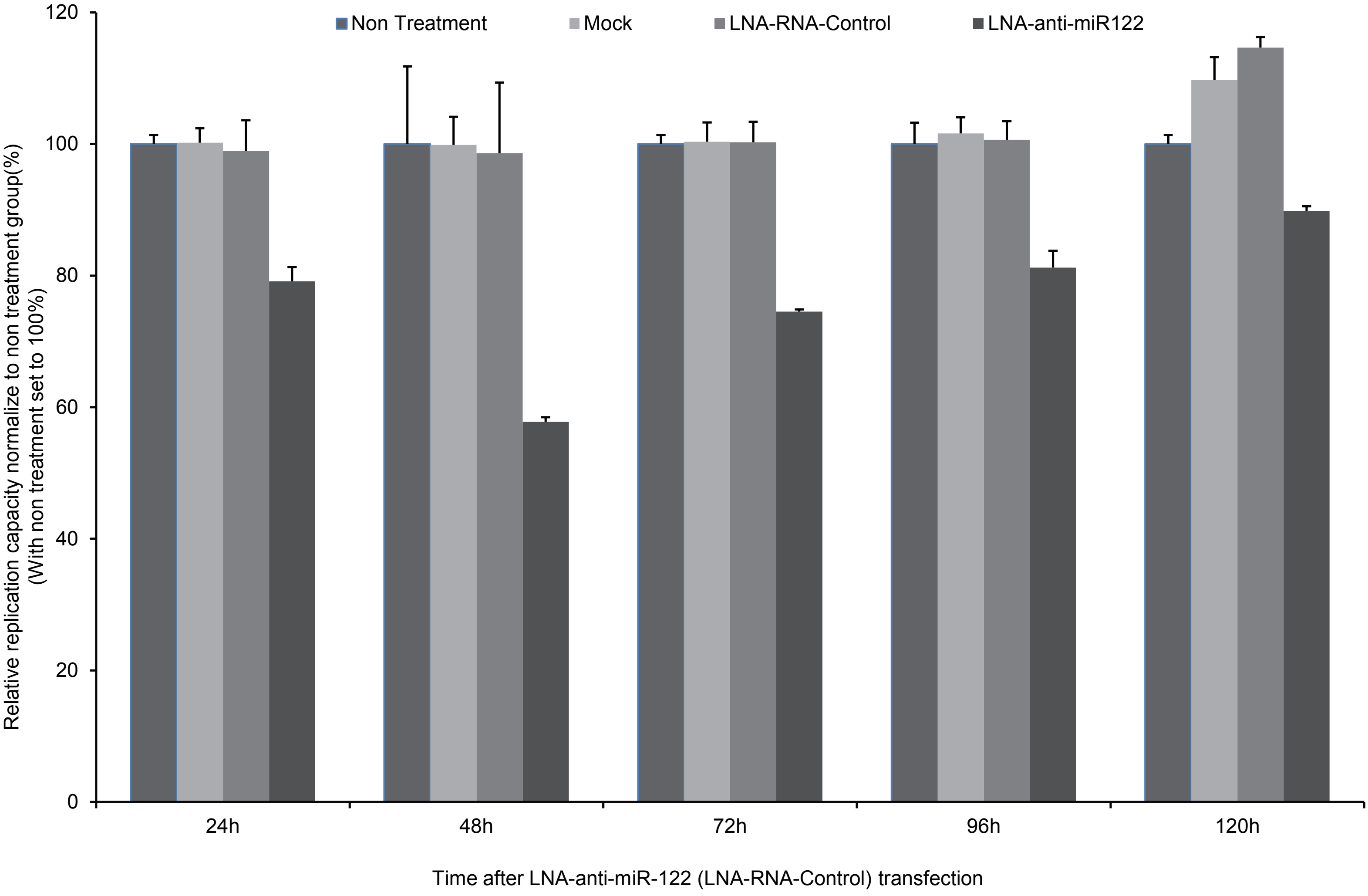

Supplementary Fig.S7

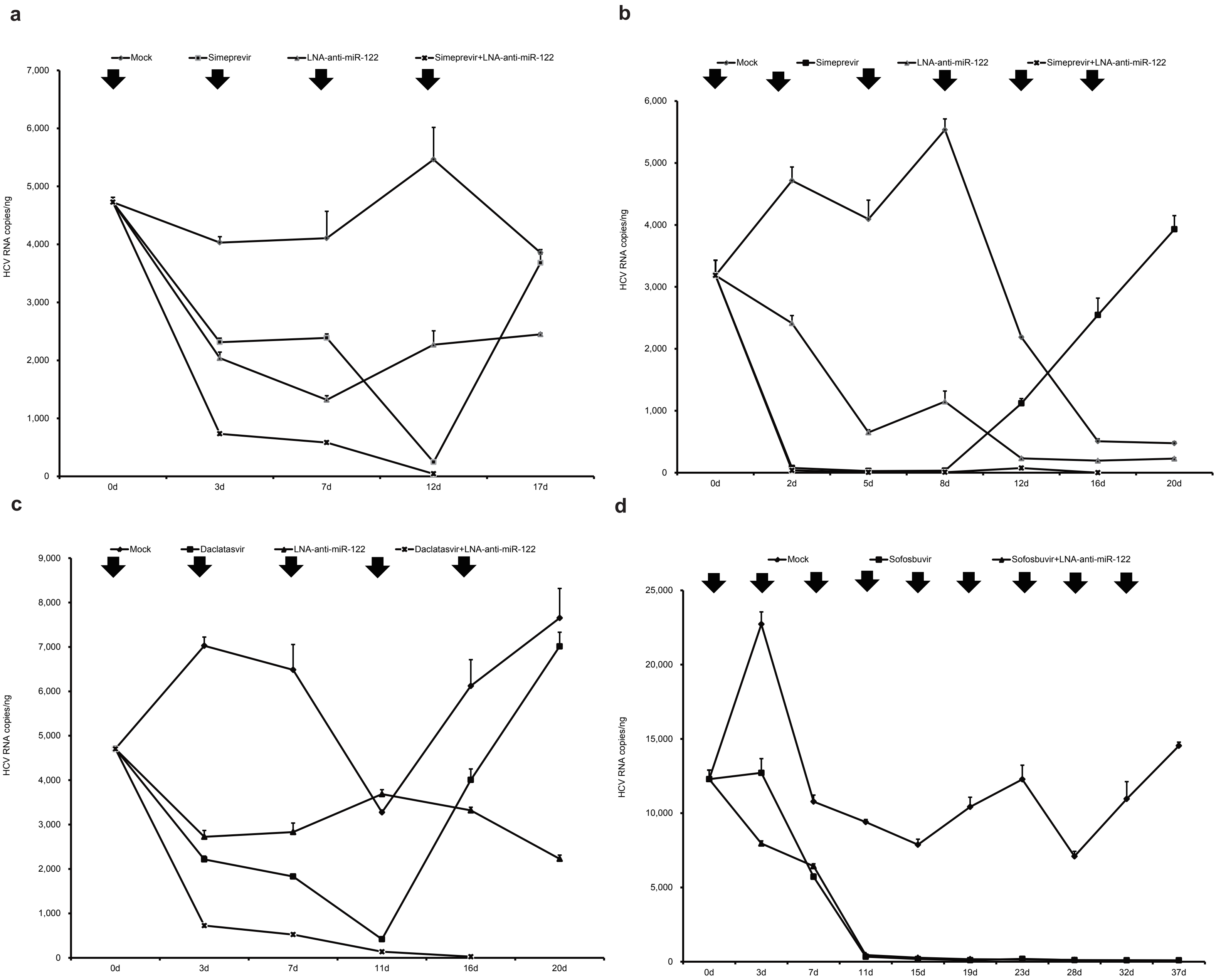

Supplementary Fig.S8

a

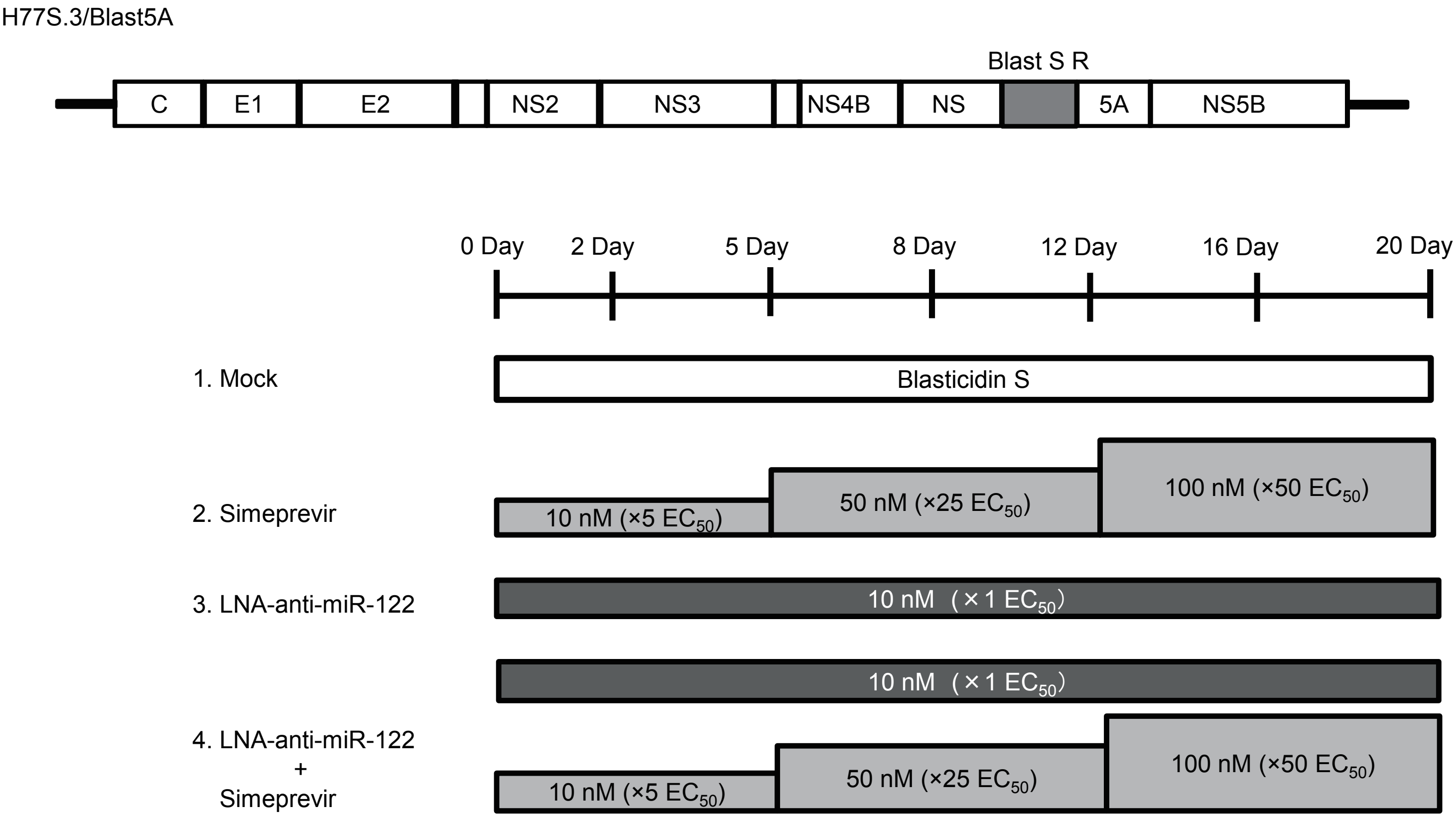

b

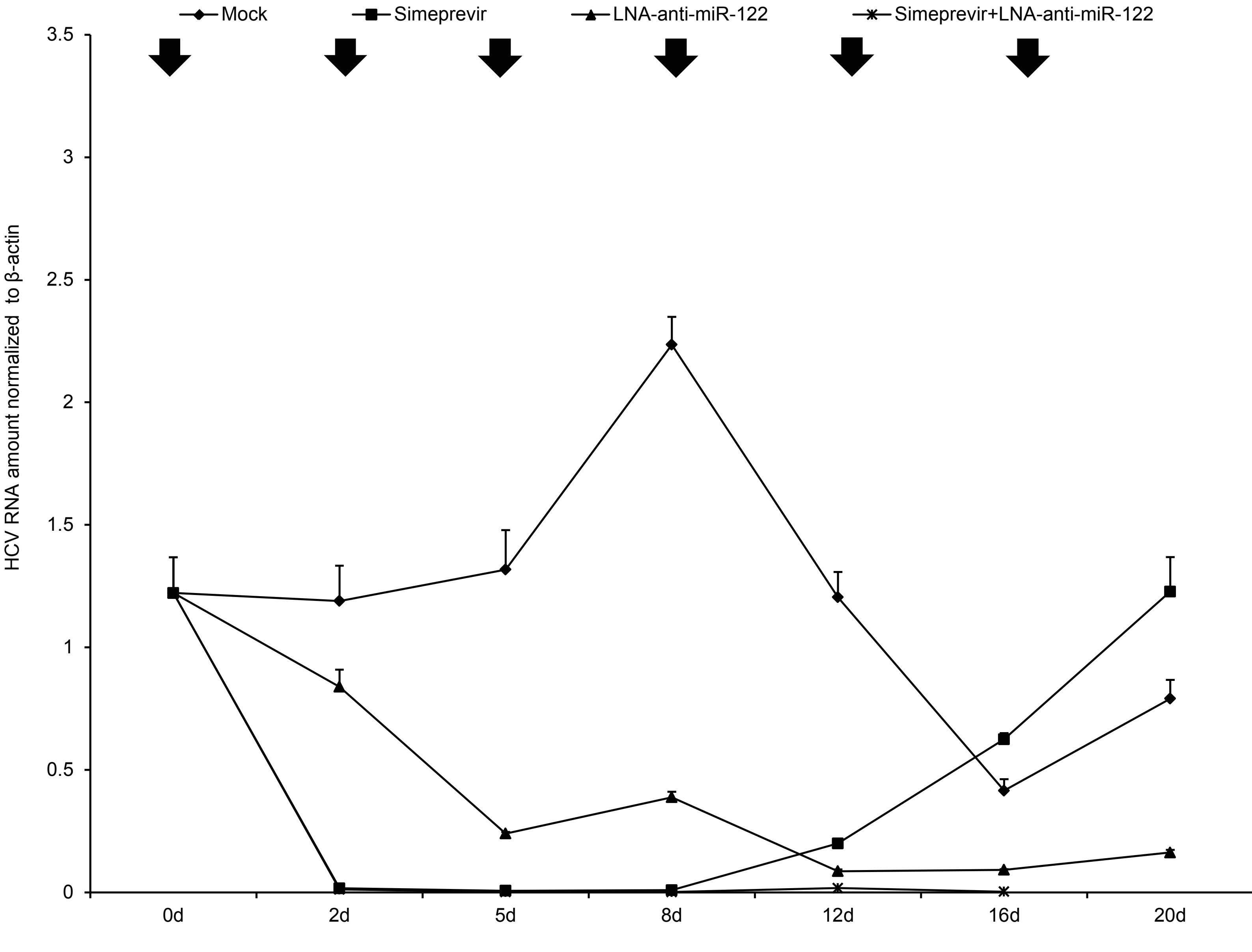

Supplementary Fig.S9

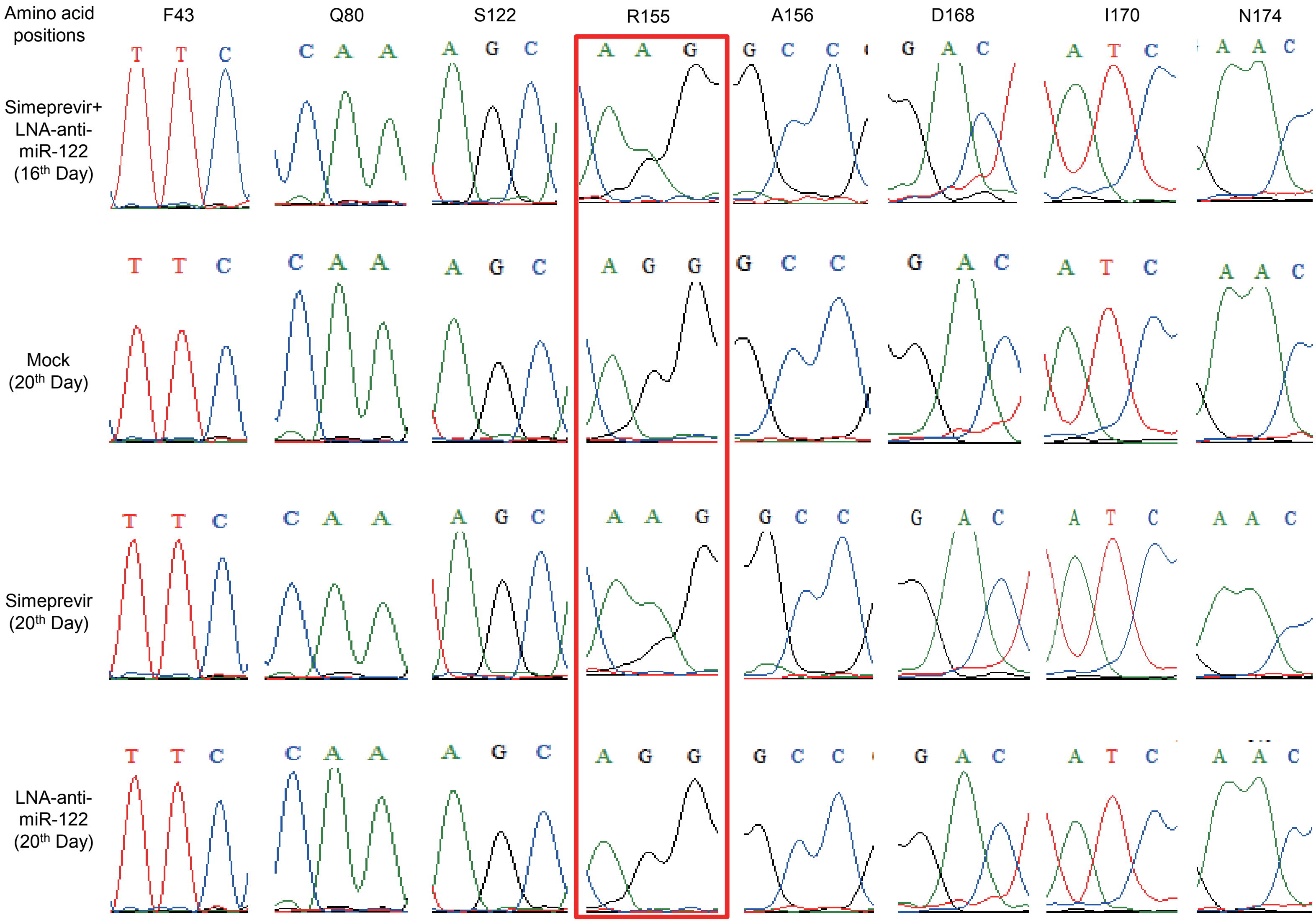

Supplement: Supplementary Information [file srep30939-s1.pdf]
